# “Nanoskeleton” Si-SiO_*x*_/C Anodes toward Highly Stable Lithium-Ion Batteries

**DOI:** 10.1021/acsami.4c18254

**Published:** 2025-02-04

**Authors:** Xiang Guan, Yang Zhang, Ian A. Kinloch, Mark A. Bissett

**Affiliations:** National Graphene Institute, Henry Royce Institute, and Department of Materials, University of Manchester, Manchester M13 9PL, U.K.

**Keywords:** CNTs, silicon anodes, SiO_*x*_ anodes, lithium ion batteries, molecular cross-linking

## Abstract

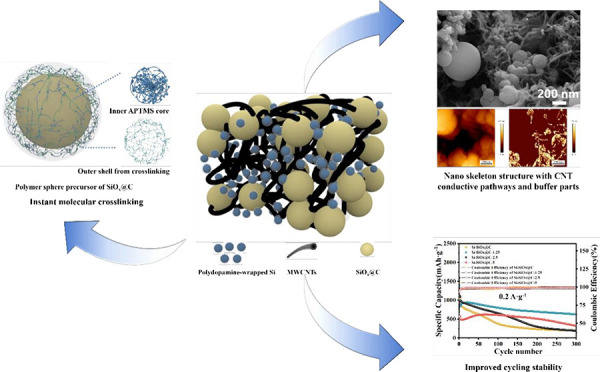

A fragile solid-electrolyte interphase (SEI) layer due to the volume expansion of silicon cannot sufficiently prevent side reactions and electrolyte consumption and restricts the application of silicon anodes in lithium-ion batteries with high cycling stability. Herein, a carbon nanotube (CNT) supported “nanoskeleton” structure with robust mechanical properties and improved conductive pathways is designed by twining CNTs with *in situ* grown SiO_*x*_/C and carbon-wrapped Si nanoparticles. The CNT “nanoskeleton” can improve electrical contact between particles, promoting the formation of a denser and more homogeneous SEI layer. Moreover, the buffer region granted by the CNTs can tolerate the volume expansions of Si, avoiding the repeated destruction of the SEI layer during the continuous lithiation and delithiation processes. Combined with these advantages, the anode with optimal CNT content can deliver both a high capacity (918 mAh·g^–1^ at 200 mA·g^–1^) and high-capacity retention (74% after 300 cycles) with relieved volume expansion (71.4%). The capacity of the NMC111 full cell with the synthesized Si-SiOx/C anode is retained at 71 mAh·g^–1^ after 500 cycles at 100 mAh·g^–1^ with a capacity retention of 72%.

## 1 Introduction

The demand for efficient energy storage technology for electronic devices and electric vehicles, such as long cycle life and high energy densities, is continually increasing. Lithium-ion batteries (LIBs) play a key role in this field. However, it remains a great challenge to design LIBs with ever higher capacity and stable cycle life.^1,2^ Limited by the theoretical capacity of commonly used anodes (e.g., graphite: 372 mAh·g^–1^), it is difficult to meet the increasing application demands.^3^ Silicon-based anode materials attract widespread research interest due to silicon’s high theoretical capacity (3579 mAh·g^–1^), which is much higher than commercialized graphite anodes.^4^ However, the main obstacle of implementing silicon anodes into applications is the volume expansion during the charging and discharging processes (about 400%). This causes serious problems in anodes, such as pulverization of Si particles, high interfacial resistance, and continuous liquid electrolyte consumption caused by repeated SEI formation.^5^ The low Coulombic efficiency (CE) and SEI accumulation gradually increase the interfacial resistance, leading to performance decay. Also, the low conductivity of Si particles affects the battery performance.^6,7^

To suppress the volume expansion and overcome this restriction, various strategies have been attempted. Nanosized Si, like silicon nanotubes,^8,9^ silicon nanoparticles,^10^ and nanowires,^11,12^ have been shown to effectively accommodate the volume change during cycling. Liu et al.^13^ obtained nanostructured silicon particles from rice husks via thermal decomposition and magnesiothermic reduction treatment, which delivered high reversible capacity (2790 mAh·g^–1^) and long cycle life (86% capacity retention over 300 cycles at 2.1 A·g^–1^). Using rice husks as the raw material provides a green and low-cost strategy to synthesize silicon on a large scale. To improve the electrical conductivity of silicon anodes, various Si-containing composite structures have been exhibited to further improve the performance of these anodes,^14,15^ which provide buffer regions for volume expansion by surface modification of silicon^16^ or design of different carbon-based structures,^17^ including carbon nanotubes^18^ and graphene.^5^ Also, these methods are intended to reduce the direct exposure of Si with the liquid electrolyte to minimize side reactions, improve electrical conductivity, and further increase cycling life. However, the carbon coating layer alone has less of an interfacial interaction with the silicon surface and is easily cracked, and higher contents of carbon could reduce the specific capacity per gram. To solve this problem, combinations of carbon and silicon oxide (SiO_*x*_, 0 < *x* < 2) have been designed and prepared with different methods. SiO_*x*_ is a trade-off alternative of Si anodes, which has lower capacity (2200–3580 mAh·g^–1^) but longer cycling life.^19^ The lithium silicates and Li_2_O are formed during the initial lithiation cycle of SiO_*x*_, which deposits on the outer layer of Si particles to form a stable SEI layer and buffer the volume expansion to some degree.^20^ Incorporating SiO_*x*_ with Si/C composite materials, the obtained composites can improve cycling stability without sacrificing too much capacity. Hernandha et al.^21^ coated Si particles with a SiO_*x*_ layer via interaction with supercritical CO_2_ and deposited a carbon film on the outer layer using glucose as the precursor. The obtained anodes showed a high specific capacity of 918 mAh·g^–1^ at a rate of 5 A·g^–1^. Halim et al.^22^ synthesized a SiO_*x*_/C matrix from the thermal conversion of polysiloxanes. The structure of SiO_*x*_/C is similar to SiO_*x*_, and the free carbon inside particles could offer the particles with better electronic conductivity, better rigidity, and cycling performance.^23,24^ To take advantage of both Si and SiO_*x*_/C, embedding Si in a matrix of SiO_*x*_/C should be a feasible and reliable strategy.^25^

Herein, we present a “nanoskeleton” structured Si-SiO_*x*_/C composite anode featuring an improved mechanical buffer and electrical contact between nanoparticles upon cycling, enhancing the battery performance. For material fabrication, due to the interaction between glutaraldehyde and polydopamine layers in the precursor solution, the fast and homogeneous cross-linking reaction could rapidly form SiO_*x*_/C precursors around polydopamine-wrapped silicon (PDA-Si) with conductive multiwall carbon nanotubes (CNTs) as the skeleton, once (3-aminopropyl)trimethoxysilane (APTMS) was added dropwise. After carbonization, the CNTs were tightly packed between the PDA-Si and SiO_*x*_/C nanoparticles. Due to the particle-stacked structure, the curled CNTs can provide flexible springs to ensure reversible buffers for volume expansion during cycling. Meanwhile, this buffering “nanoskeleton” structure can provide adequate conductive pathways between carbon-containing SiO_*x*_/C and carbon-wrapped Si derived from PDA-Si. Promisingly, in this CNT-supported Si-SiO_*x*_/C structure (named as Si-SiO_*x*_/C-1.25, Si-SiO_*x*_/C-2.5, and Si-SiO_*x*_/C-5, respectively, which represent the mass ratio of CNTs inside), the continuous electron transfer and relief of internal stress caused by Si during the lithiation and delithiation processes could be realized simultaneously. As a result, the nanoskeleton supported Si-SiOx/C-1.25 anodes deliver a high capacity (918 mAh·g^–1^ at 200 mA·g^–1^) and high-capacity retention (74% after 300 cycles) with relieved volume expansion (71.4%). Confirmed by *in-operando* XRD and XPS of fully lithiated anodes, the stable and dense SEI layers are formed on the surface of CNT-supported nanoskeleton structure, providing high cycling stability. Paired with NMC111, the specific capacity of the full cell can be stable at about 71 mAh·g^–1^ after 500 cycles at 100 mAh·g^–1^.

## 2 Experimental Section

### 2.1 Materials

Dopamine hydrochloride (Sigma-Aldrich), silicon nanopowder (US Research Nanomaterials, >98%, 30–50 nm), tris(hydroxylmethyl)aminomethane (Tris buffer, Sigma-Aldrich, ≥99.8%), hydrochloric acid (Sigma-Aldrich, 37%), carbon nanotubes (Sigma-Aldrich, multiwalled), glutaraldehyde solution (Sigma-Aldrich, C_5_H_8_O_2_, GA, 50 wt % in H_2_O), (3-aminopropyl)trimethoxysilane (Sigma-Aldrich, APTMS, 97%), Super P (Alfa Aesar, 99+%), sodium alginate (Sigma-Aldrich), LiNi_1/3_Mn_1/3_Co_1/3_O_2_ (NMC111, Sigma-Aldrich, >98%), and PVDF (Arkema, Kynar HSV900) were used as received. The electrolyte for the silicon–lithium half-cell was prepared by dissolving 5 wt % fluoroethylene carbonate (FEC) in 1.0 M LiPF_6_ in ethylene carbonate (EC):diethyl carbonate (DEC):dimethyl carbonate (DMC) 1:1:1 (v/v/v). The electrolyte for the NMC111 full cell was 1.0 M LiPF_6_ in ethylene carbonate (EC):diethyl carbonate (DEC):dimethyl carbonate (DMC) 1:1:1 (v/v/v).

### 2.2 Synthesis of Polydopamine Wrapped Silicon Nanoparticles

This synthesis procedure is modified based on this literature.^26^ First, 400 mg of tris(hydroxylmethyl)aminomethane was dissolved in 200 mL of DI water, and subsequently HCl was added to adjust the pH to 8.5 as a buffer solution. Then, silicon nanopowders (1.25 g) were dispersed in the above solution via bath sonication for 10 min. Dopamine hydrochloride (200 mg) was added, and the mixture was stirred for 24 h. The polydopamine wrapped silicon nanoparticles are collected by centrifugation and washed three times with DI water. Followed by drying in oven for 8 h, the final product is abbreviated as PDA-Si.

### 2.3 Synthesis of Si-SiO_*x*_/C-CNTs

CNTs and PDA-Si (150 mg) were dispersed in GA solution (1%, 40 mL) via bath sonication for 30 min and stirring overnight. Then 0.746 mL of APTMS was added dropwise into the above suspension, followed by stirring for 30 min. After filtration and washing, Si-SiO_*x*_/C-CNTs are obtained by freeze-drying and carbonization (700 °C, 2 h, Ar atmosphere). Based on the added amount of CNT (0, 4.6, 9.2, and 18.4 mg), the carbonized products (Si-SiO_*x*_/Cs) are abbreviated as Si-SiO_*x*_/C, Si-SiO_*x*_/C-1.25, Si-SiO_*x*_/C-2.5 and Si-SiO_*x*_/C-5, respectively, based on the mass ratio of CNT inside, while the uncarbonized samples (P-Si-SiO_*x*_/Cs) are abbreviated as P-Si-SiO_*x*_/C, P-Si-SiO_*x*_/C-1.25, P-Si-SiO_*x*_/C-2.5 and P-Si-SiO_*x*_/C-5, respectively. Also, the carbonized particle from PDA-Si is abbreviated as C-PDA-Si.

### 2.4 Preparation of Electrodes

Anodes were prepared by thoroughly grinding Si-SiO_*x*_/C materials, Super P (carbon black), and sodium alginate with the mass ratio of 8:1:1 in a mortar and then dispersed in deionized water via speed mixer. The above slurry was coated onto copper foil via doctor blade with the thickness of 200 μm. After vacuum drying at 90 °C overnight, the anodes are punched into discs (Φ = 12 mm) and then transferred into glovebox for use. The loading of the active materials is around 1.5–2.0 mg·cm^–2^. For NMC111 cathodes, NMC111 was ground with Super P in a mortar and poured into PVDF solution (mass ratio of NMC111:Super P:PVDF = 8:1:1). After stirring overnight, the slurry was spread on the aluminum foil with the thickness of 100 μm and dried at 90 °C overnight under vacuum. Then the electrodes are punched into discs (Φ = 10 mm) with the loading of active materials around 2.5 mg/cm^2^.

### 2.5 Characterization

The Fourier-transform infrared spectroscopy (FT-IR) spectra of uncarbonized samples were collected by a Nicolet 7800 spectrometer with ATR. The crystal structures of Si-SiO_*x*_/C-CNTs are characterized by X-ray diffraction (XRD) using a Bruker D8 X-ray diffractometer (40 kV, 100 mA) with a copper target and Ni filter. The carbon framework of Si-SiO_*x*_/C-CNTs were verified by a Raman spectrometer (inVia-Reflex, Renishaw, U.K.) with a laser of 514 nm. The surface areas of the Si-SiO_*x*_/C-CNTs were obtained by N_2_ adsorption and desorption isotherms via QUADRASORB evo with nitrogen as adsorbate and liquid nitrogen as cooling medium. The variance in uncarbonized, carbonized Si-SiO_*x*_/C-CNTs and components of the SEI layer were verified by XPS analysis using a Kratos Axis Ultra DLD spectrometer (Kratos Analytical) with a monochromatic Al Kα source (1486.6 eV). The morphologies of Si-SiO_*x*_/C-CNTs and anodes were analyzed by SEM (Zeiss, Sigma VP), TEM and STEM (Thermo fisher, Talos F200X). Morphologies and Young’s modulus of anodes were measured by atomic force microscopy (AFM) via a JPK Nanowizard. Conductive pathways of the anodes were recorded by conductive AFM (CAFM) with PPP-EFM probes.

For in-operando testing, specific split cells were prepared. The in-operando XRD cell contains an X-ray transparent beryllium window that allows diffracted X-rays to penetrate (EQ-STC-BEW, MTI Corporation). The XRD patterns were acquired simultaneously at a fixed time interval (20 min) during the discharge process. Based on the discharge profile and recording time, the XRD pattern at selected voltages can be obtained. The in-operando optical cell consists of a quartz observation window for cross-section imaging (EQ-STC-PQW, MTI Corporation). The images were recorded at a fixed time interval (20 min) simultaneously during the discharge process. Based on the discharge profile and recording time, cross-sectional images of the electrodes can be recorded. All in-operando tests were conducted under a current density of 10 mA·g^–1^ with lithium metal as the counter electrode. The data were all recorded at different voltages during the initial lithiation process, including open circuit voltage (OCV), 1.5, 0.8, 0.3, 0.2, 0.1, and 0.005 V.

### 2.6 Electrochemical Analysis

Electrochemical impedance spectroscopy (EIS) of half cells before and after fixed cycles was conducted within the frequency range of 0.1 Hz to 1 MHz with 0.01 V of amplitude. Cyclic voltammetry (CV) under different scan rates (0.1, 0.3, 0.5, 0.7, and 1 mV/s) were recorded. EIS and CV data were collected using Ivium CompactStat. The cycling performance and stability of Si-SiO_*x*_/Cs are collected using a Landt battery tester (CT2001A) under various current density between 0.005 and 2.5 V versus Li/Li^+^ at room temperature with lithium metal as both counter and reference electrode. Li^+^ diffusion coefficient of the anodes was determined by the galvanostatic intermittent titration technique (GITT) with a titration time and rest time of 10 min and 2 h at 100 mA·g^–1^, respectively. Si-SiO_*x*_/Cs/electrolyte/NMC111 full cell was cycled between 3.0 and 4.3 V under 100 mA·g^–1^ with the prelithiation process of Si-SiO_*x*_/Cs. The prelithiation was started by assembling the half cells with lithium metal and Si-SiO_*x*_/Cs as electrodes. After the initial full lithiation process at 100 mA·g^–1^, the prelithiated Si-SiO_*x*_/Cs anode can be obtained by disassembly of the half-cell and further used for full cell assembly. Before pairing the full cell, the N/P ratio should be calculated by the capacity ratio between the anode (the negative electrode) and cathode (the positive electrode) and kept between 1.1 and 1.2 by adjusting the loading of anodes and cathodes. All batteries were assembled in an Ar-filled glovebox with oxygen content less than 0.5 ppm and H_2_O content less than 0.5 ppm.

## 3 Results and Discussion

**Scheme 1:**
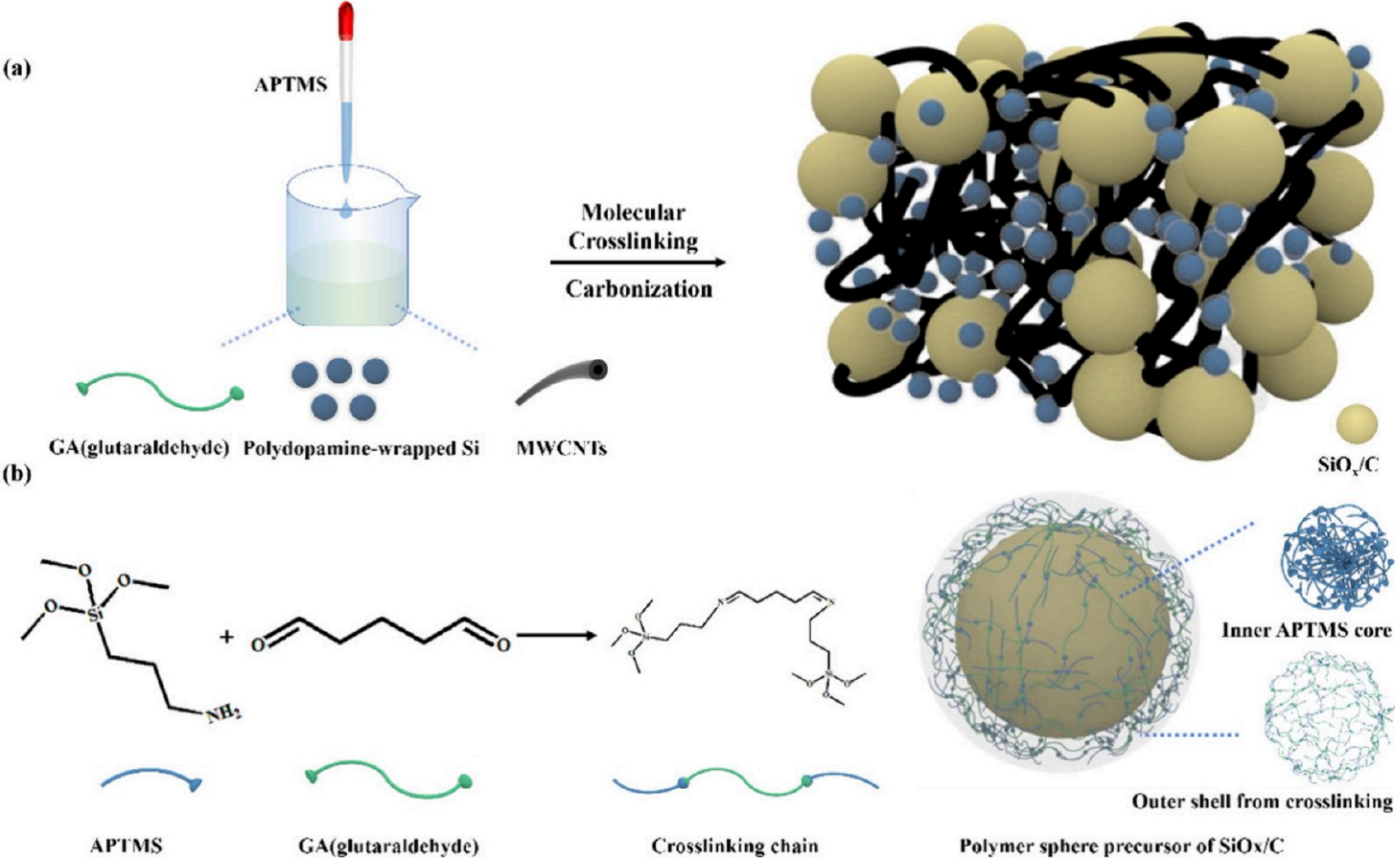
(a) Illustration of the Synthetic Procedure of CNT Supported Nanoskeleton Si-SiO_*x*_/Cs and (b) Reaction Equation of Crosslinking between APTMS and GA

**Figure 1:**
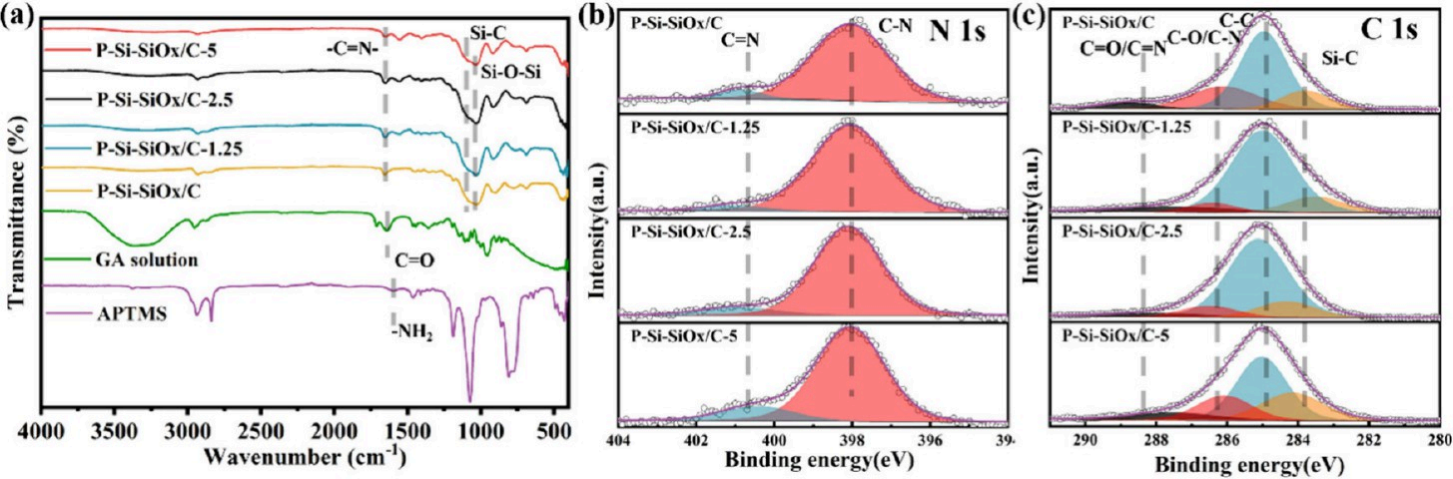
(a) FT-IR spectra of P-Si-SiO_*x*_/Cs, GA and APTMS; (b, c) N 1s and C 1s XPS spectra of P-Si-SiO_*x*_/Cs.

To fabricate the CNT “nanoskeleton” structured Si-SiO_*x*_/C, shown schematically in Scheme 1, the CNTs and polydopamine-wrapped silicon nanoparticles (PDA-Si) were first dispersed in GA solution via sonication and overnight stirring. When the added APTMS is dispersed into fine oil droplets under vigorous stirring, the droplets can react with GA molecules to obtain the precursor of SiO_*x*_/C with the outer polymer shell. The simple cross-linking reaction between GA and APTMS *in situ* produces the polymer spheres based on the CNTs with Si nanoparticles filling in the structure. After freeze-drying and carbonization, the obtained powders exhibit the twined 3D structure seen microscopically. The existence of the polydopamine layer outside the silicon nanoparticles has been verified by FT-IR and Raman (Figure S1). The FT-IR of dopamine chloride in Figure S1a shows sharp peaks at 1612 and 3332 cm^–1^ related to amine (N–H) groups.^27^ For PDA-Si, the detected peaks at 1515 and 1656 cm^–1^ are from the indole unit of the PDA structure. It can be seen that C–O (1012 cm^–1^) and C–N (1181 cm^–1^) stretching vibrations as well as −C–C–H (1284 cm^–1^) bending bonds in dopamine chloride disappeared or diminished upon polymerization to the PDA layer. The interplane bending bonds of the H–N–H group in dopamine, which appeared at 813 and 1612 cm^–1^, were weakened, verifying PDA formation. For Raman in Figure S1b, the peaks at 1356 and 1585 cm^–1^ were related to the deformation of catechol, further confirming the PDA layer over Si.^28^ Before carbonization, the chemical structures of P-Si-SiO_*x*_/C, P-Si-SiO_*x*_/C-1.25, P-Si-SiO_*x*_/C-2.5, and P-Si-SiO_*x*_/C-5 were characterized by FT-IR (Figure 1a). The stretching peaks of −C=N– in SiO_*x*_/C precursors were located at 1657 cm^–1^, verifying the successful reaction between C=O from GA (1647 cm^–1^) and −NH_2_ groups (1605 cm^–1^) from APTMS after cross-linking reaction.^29,30^ The peaks from 1000 to 1200 cm^–1^ are ascribed to Si–C and Si–O–Si bonds, providing the silicon oxide source after carbonization.

To verify the chemical valence states of P-Si-SiO_*x*_/Cs, XPS analysis was conducted (Figure S2). In Figure 1b, the N 1s core level spectra of P-Si-SiO_*x*_/C, P-Si-SiO_*x*_/C-1.25, P-Si-SiO_*x*_/C-2.5, and P-Si-SiO_*x*_/C-5 exhibit similar peaks from 395 to 403 eV, which can be attributed to C=N (400.8 eV) and C–N (398.1) peaks.^31^Figure 1c presents the C 1s spectra, in which the four peaks at around 283.8, 285.0, 286.2, and 288.2 eV are assigned to Si–C, C–C, C–O/C–N and C=O/C=N, respectively.^32^ Si 2p spectra showed Si–O and Si–C peaks at 101.1 and 102.2 eV (Figure S3). Combining with the typical peak of Si–O bond in the O 1s spectra (Figure S4), the XPS results are in accordance with the FT-IR, confirming the successful cross-linking reaction.

**Figure 2:**
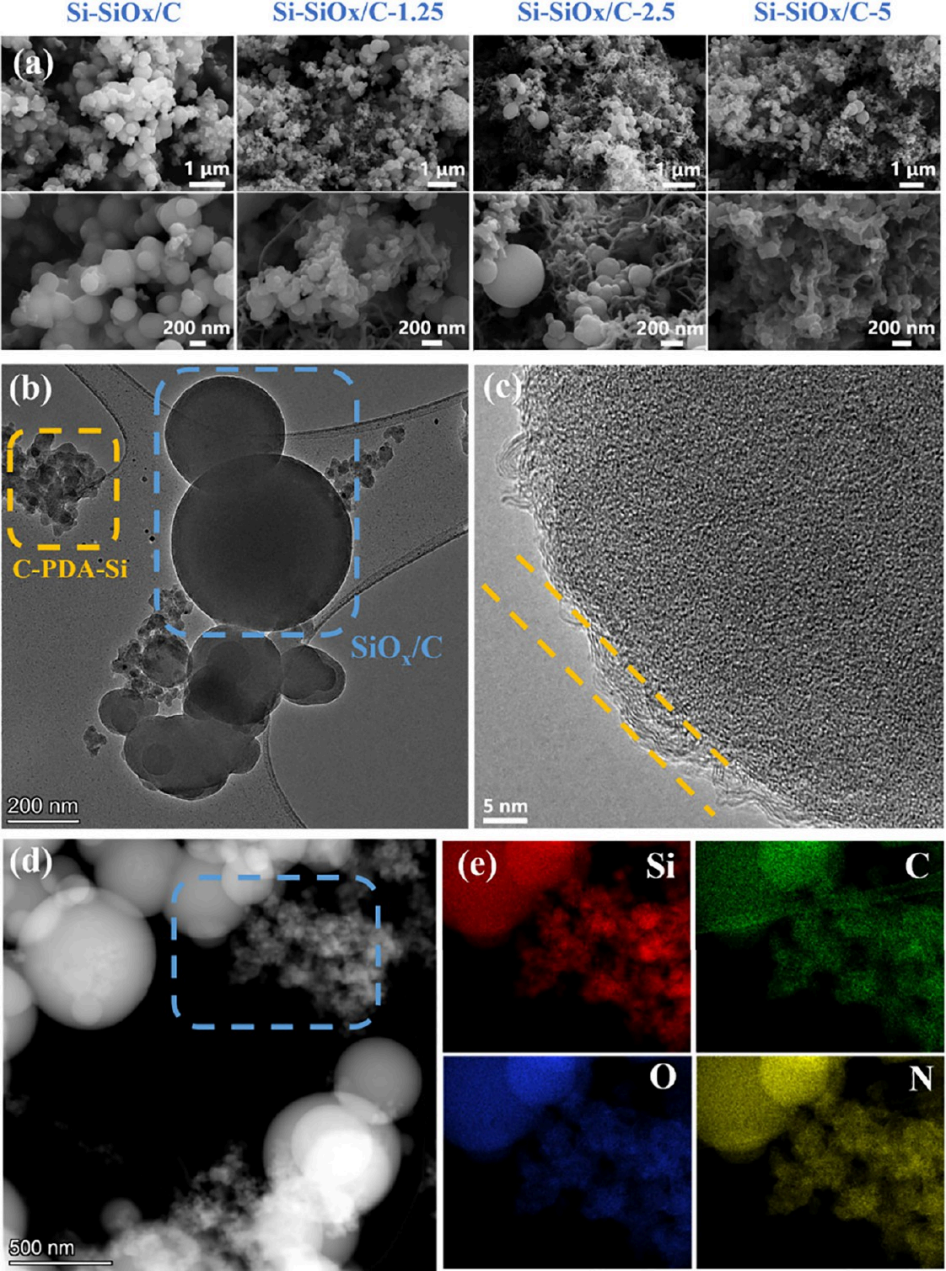
(a) SEM images of Si-SiO_*x*_/Cs; (b) TEM images of Si-SiO_*x*_/C-1.25; (c) HR-TEM image of C-PDA-Si; (d, e) STEM image and related EDS mapping of Si-SiO_*x*_/C-1.25.

After carbonization, the detailed morphologies of Si-SiO_*x*_/Cs were revealed by SEM. As shown in Figure 2a, the larger spheres are SiO_*x*_/C particles carbonized from the reaction products between GA and APTMS, while the smaller spheres are C-PDA-Si carbonized from PDA-Si. Si-SiO_*x*_/C shows a mixture of C-PDA-Si and SiO_*x*_/C, while Si-SiO_*x*_/C-1.25, Si-SiO_*x*_/C-2.5, and Si-SiO_*x*_/C-5 exhibit twined-CNTs-supported structure, in which the SiO_*x*_/C particles depend on CNTs to form with C-PDA-Si filled in. Based on our previous work, the spherical structure of SiO_*x*_/C is granted by the cross-linking reaction between GA and APTMS with GA as cross-linkers.^33^ To confirm this, the morphology of dried APTMS without adding GA is shown in Figure S5, which is a flake-like structure rather than a spherical structure.

The fine structure and elemental composition of the Si-SiO_*x*_/Cs were further characterized by TEM and STEM-EDS. Figure 2b shows the TEM image of Si-SiO_*x*_/C-1.25, in which the diameter of SiO_*x*_/C ranges from 200 to 400 nm and the diameter of C-PDA-Si is about 30–50 nm. The 3–5 nm carbon layer outside the C-PDA-Si was exhibited in Figure 2c, which is derived from the carbonization of the polydopamine layer. The high-angle annular dark-field scanning transmission electron microscopy (HAADF-STEM) image of the curly CNTs inside the sample is shown in Figure S6 with a diameter of about 20 nm. Furthermore, the corresponding elemental composition of Si-SiO_*x*_/C-1.25 has been shown. Figure 2e illustrates the HAADF-EDS image of the circled region in Figure 2d. SiO_*x*_/C is composed of Si, C, O, and N homogeneously, which is attributed to the fast-cross-linking reaction. EDS of C-PDA-Si showed comparable results, in which C and N are derived from the polydopamine layer. Also, the twined structure between CNT and C-PDA-Si is shown in Figure S7 with uniform elemental distribution. Based on the SEM and TEM results, the CNTs supported “nanoskeleton” structure has been clearly revealed, which is expected to improve the mechanical property and produce more conductive pathways.

**Figure 3:**
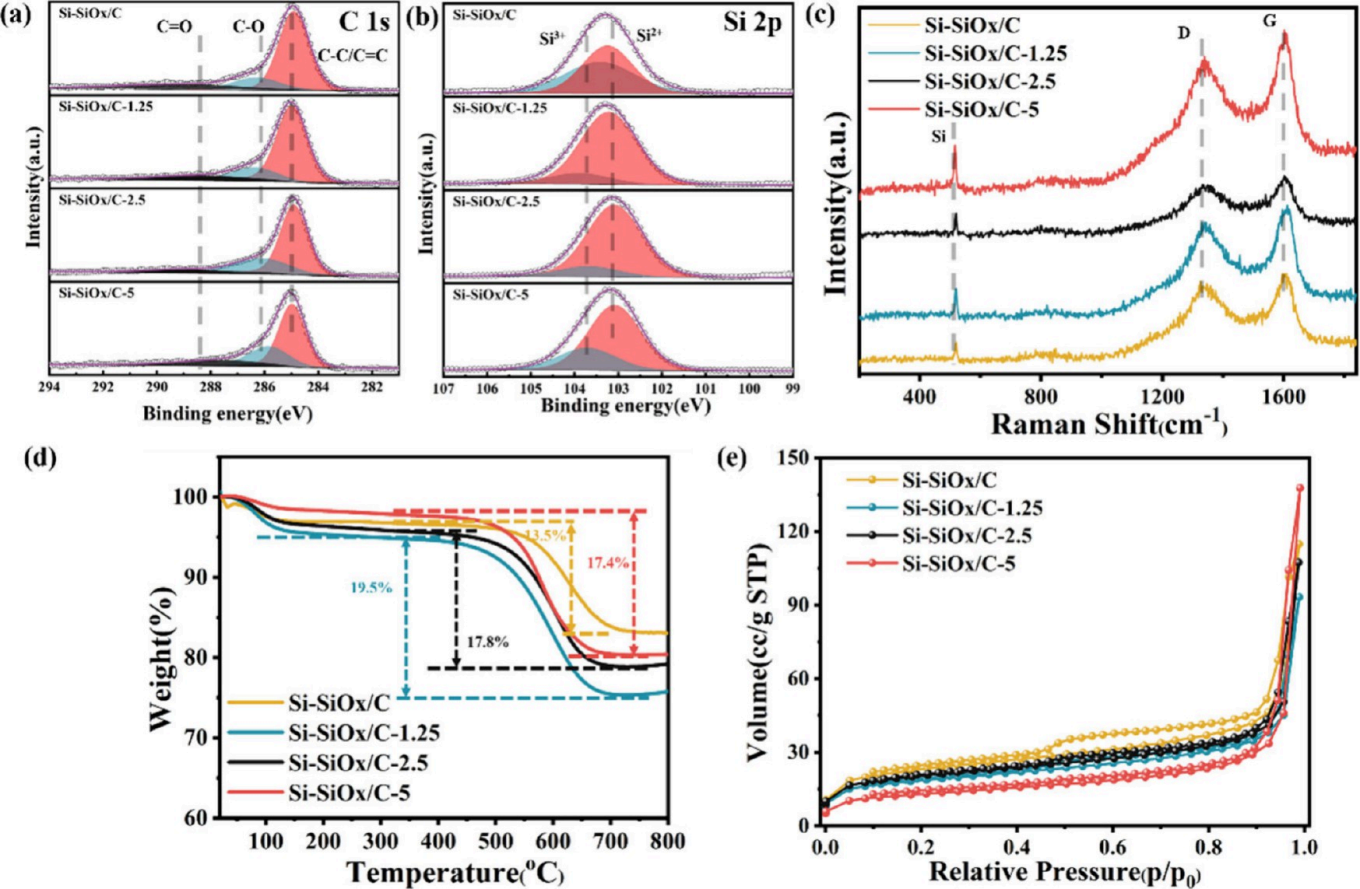
(a, b) C 1s and Si 2p XPS spectra of Si-SiO_*x*_/Cs; (c) Raman spectra of Si-SiO_*x*_/Cs; (d) TGA curves of Si-SiO_*x*_/Cs under air from 20 to 800 °C with ramp rate of 10 °C·min^–1^; (e) N_2_ adsorption–desorption isotherms of Si-SiO_*x*_/Cs.

During the carbonization process, the polymer chains are broken and transformed into SiO_*x*_/C and C-PDA-Si particles. XPS analysis was conducted to investigate the chemical valence state changes in Si-SiO_*x*_/Cs in Figure S8. From Figure 3a, the C 1s spectra show three peaks at 284.8, 285.8, and 288.3 eV, which are assigned to the C–C/C=C, C–O, and C=O bonds, respectively. Compared with C 1s of P-Si-SiO_*x*_/C (Figure 1c), the chemical bonds between carbon and nitrogen or silicon disappeared after carbonization, showing the transformation from polymer chains to amorphous carbon. This could also be verified by XRD (Figure S9), in which the broad peak from 15° to 30° is ascribed to amorphous carbon and silicon oxide and the series of sharp peaks are from crystalline silicon.^34^ In Figure 3b, the broad peaks from 101 to 106 eV could be fitted with Si^2+^ (103.1 eV) and Si^3+^(103.7 eV).^35^ The typical peak of the Si–O bond at around 532 eV is also confirmed in the O 1s spectra (Figure S10). For the Si 2p spectrum of Si-SiO_*x*_/C, the normalized percentages of Si^2+^ and Si^3+^ are 48.1% and 51.99%, respectively. Therefore, the estimated Si valence state is 3.56. Similarly, the Si valence states of Si-SiO_*x*_/C-1.25, Si-SiO_*x*_/C-2.5, and Si-SiO_*x*_/C-5 are 2.14, 2.14, and 2.28, respectively (Table S1). The valence differences between samples with and without CNTs might be caused by the existence of CNTs having an effect on the formation of SiO_*x*_/C polymer precursors. The rough carbon, silicon, and oxygen contents of Si-SiO_*x*_/C with CNTs based on XPS can be summarized in Table 1, showing that higher CNT contents bring higher Si contents. It means that the added CNT with high surface area can help the reaction between APTMS and GA. The O/Si ratios are all around 1.95. The ratios of D and G bands of Si-SiO_*x*_/Cs in the Raman spectra are 0.895, 0.854, 0.872, and 0.897, respectively (Figure 3c), indicating the formation of a carbon framework during the carbonization process.

**Table 1:** C, Si, O Content in Si-SiO_*x*_/Cs with CNTs

	C (atom %)	O (atom %)	Si (atom %)	O/Si
Si-SiO_*x*_/C-1.25	28.89	46.4	23.71	1.957
Si-SiO_*x*_/C-2.5	25.11	49.52	25.37	1.952
Si-SiO_*x*_/C-5	19.76	53.09	27.15	1.955

Moreover, the carbon content was analyzed by TGA under air atmosphere from 20 to 800 °C with the ramp rate of 10 °C/min (Figure 3d). Higher carbon contents can provide higher electrical conductivity and more conductive pathways. The determined carbon contents of Si-SiO_*x*_/C, Si-SiO_*x*_/C-1.25, Si-SiO_*x*_/C-2.5, and Si-SiO_*x*_/C-5 are 13.5%, 19.5%, 17.8%, and 17.4% with a decomposition temperature at about 500 °C. The rise of TGA curves after 700 °C was caused by the oxidation of Si in air under high temperature.^36^ Based on the varied feed ratios, the added CNTs did increase carbon content compared with Si-SiO_*x*_/C. However, higher CNT contents did not linearly increase the carbon content. Si-SiO_*x*_/C-1.25 exhibits the highest carbon content, which means the proper amount of CNTs can improve the reaction between GA and APTMS due to CNTs’ high surface area. This can also be verified by XPS results in Figure 3b and Table 1, showing the effect of CNTs on the reaction, and Si-SiO_*x*_/C-1.25 has the highest carbon content. The N_2_ adsorption–desorption testing results (Figure 3e) gave specific surface areas of Si-SiO_*x*_/C (22.2 m^2^·g^–1^), Si-SiO_*x*_/C-1.25 (17.2 m^2^·g^–1^), Si-SiO_*x*_/C-2.5 (19.4 m^2^·g^–1^), and Si-SiO_*x*_/C-5 (16.2 m^2^·g^–1^). With the introduction of CNTs, the twined CNTs skeleton led to a more stacked structure and reduced the surface area slightly. The relatively low specific surface areas could reduce the side reactions and SEI formation compared to the nanoparticles and porous composites with high specific surface area.^37^

**Figure 4:**
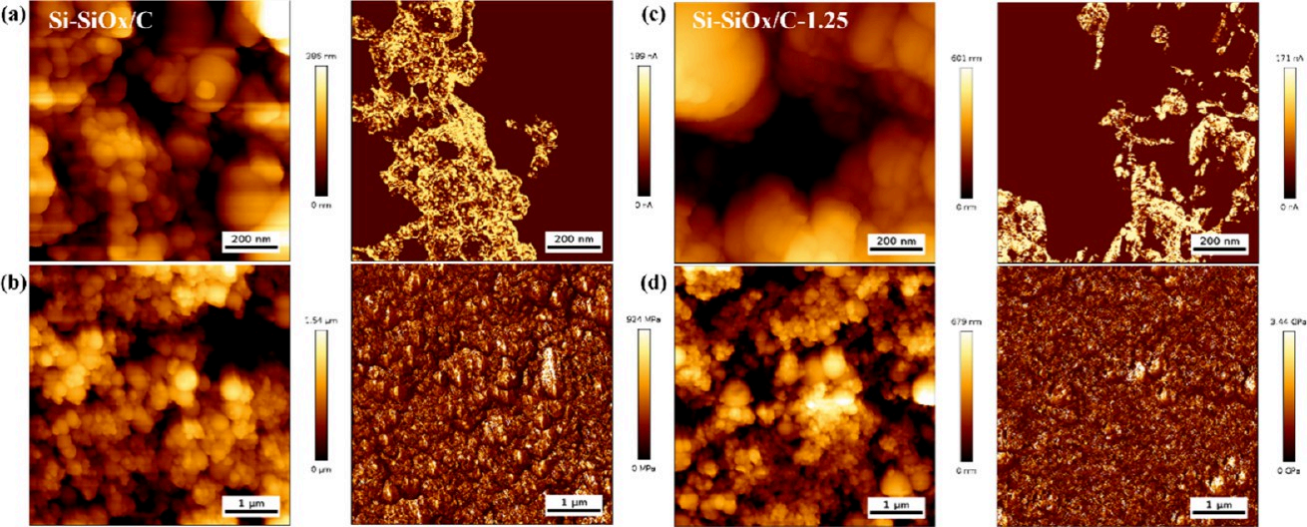
(a) AFM and CAFM images of Si-SiO_*x*_/C; (b) AFM image and Young’s modulus distribution of Si-SiO_*x*_/C; (c) AFM and CAFM images of Si-SiO_*x*_/C-1.25; (d) AFM image and Young’s modulus distribution of Si-SiO_*x*_/C-1.25.

To further investigate the increased conductive pathways and improved mechanical property granted by the CNT skeleton structure, AFM and CAFM of Si-SiO_*x*_/Cs anodes were conducted. In Figure 4a and Figure 4c, the morphologies are in accordance with the previous SEM and TEM results. Combining with the AFM morphologies, the current distribution figures clearly exhibit the conductive pathways, which is the nitrogen-doped carbon layer outside the silicon nanoparticles. The twined CNT skeleton can provide a conductive bridge between carbon-wrapped silicon particles, thus producing more conductive pathways. Si-SiO_*x*_/C-2.5 and Si-SiO_*x*_/C-5 show comparable results in Figure S11a and Figure S11c. The Young’s modulus was also obtained from AFM analysis. From Figure 4b, Figure 4d, Figure S11b, and Figure S11d, the Young’s modulus distribution was exhibited together with morphology. The maximum moduli which the anodes can reach are 924 MPa (Si-SiO_*x*_/C), 3.44 GPa (Si-SiO_*x*_/C-1.25), 2.05 GPa (Si-SiO_*x*_/C-2.5), and 1.44 GPa (Si-SiO_*x*_/C-5), respectively. The introduction of the CNT skeleton can thus improve Young’s modulus. With higher content of CNTs, the modulus gradually decreases, which is still higher than Si-SiO_*x*_/C anode without CNTs. Compared with Si-SiO_*x*_/C-1.25, the deficiency of silicon and SiO_*x*_/C particles could not fill most of the pores formed by the excessive curly CNTs in Si-SiO_*x*_/C-2.5 and Si-SiO_*x*_/C-5, providing a lower density than the nanoskeleton structure. Granted by the improved mechanical property, the volume expansion of silicon can be effectively withstood, providing a more stable SEI layer.

**Figure 5:**
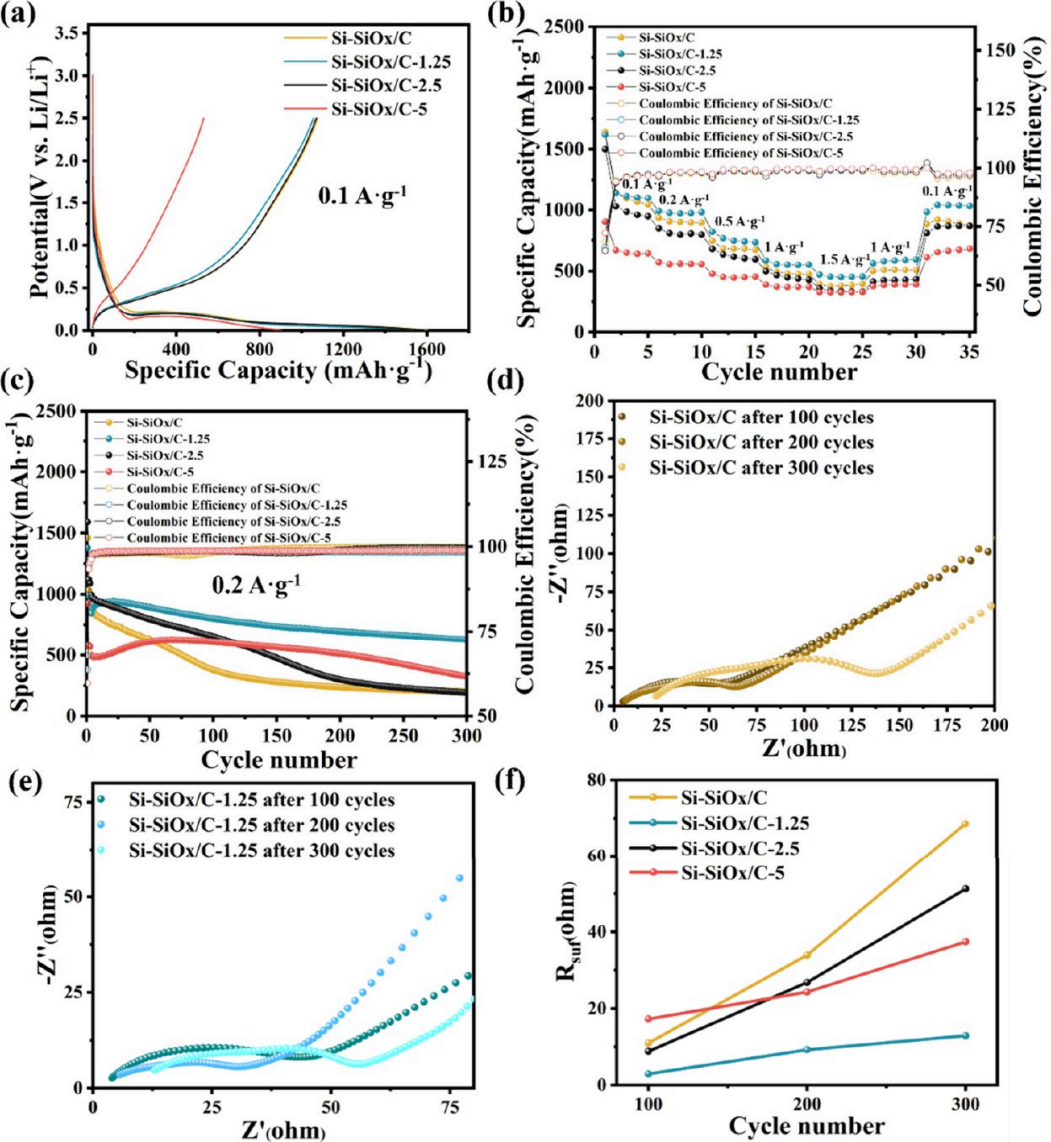
(a) Initial GCD profiles of Si-SiO_*x*_/Cs; (b) rate performance of Si-SiO_*x*_/Cs; (c) cycling performance and Coulombic efficiency of Si-SiO_*x*_/Cs at 0.2 A·g^–1^ with the first five cycles at 0.1 A·g^–1^ as activation process; (d, e) EIS spectra of Si-SiO_*x*_/C and Si-SiO_*x*_/C-1.25 after 100, 200, and 300 cycles at 0.2 A·g^–1^; (f) *R*_suf_ of Si-SiO_*x*_/Cs after 100, 200, and 300 cycles at 0.2 A·g^–1^.

The electrochemical performance of Si-SiO_*x*_/Cs anodes was investigated within the potential window from 0.005 to 2.5 V with lithium metal as the counter electrode. The initial galvanostatic charging–discharging (GCD) curves of these Si-SiO_*x*_/Cs anodes at 0.1 A g^–1^ are shown in Figure 5a. Si-SiO_*x*_/C-5 anode exhibits lower initial Coulombic efficiency (59.7%) and lower initial reversible capacity (553 mAh·g^–1^), while the other three anodes have close initial Coulombic efficiency (69%) and similar initial reversible capacity (∼1000 mAh·g^–1^). Low surface area of Si-SiO_*x*_/C-5 (Figure 3e) means it exhibits a denser structure and less inner Si access to the liquid electrolyte. Less Si involved in the electrochemical reaction caused a lower capacity compared with other Si-SiO_*x*_/Cs. Additionally, the lower Coulombic efficiency of Si-SiO_*x*_/C-5 was not solely caused by the formation of the SEI layer. During the initial cycle, the high contents of CNTs inside can also trap part of the inserted lithium ion during initial lithiation process, causing irreversible lithium ion capacity.^38,39^ The rate performance of the four anodes at the current densities from 0.1 A·g^–1^ to 1.5 A·g^–1^ was also investigated (Figure 5b). Si-SiO_*x*_/C-1.25 exhibits the highest capacity with the discharge capacity of 1116 mAh·g^–1^, 970 mAh·g^–1^, 749 mAh·g^–1^, 551 mAh·g^–1^, and 452 mAh·g^–1^ at 0.1 A·g^–1^, 0.2 A·g^–1^, 0.5 A·g^–1^, 1 A·g^–1^, and 1.5 A·g^–1^, respectively. The discharge capacity of Si-SiO_*x*_/C-1.25 is fully recovered when the current density returns to 1 A·g^–1^ (585 mAh·g^–1^) and 0.1 A·g^–1^ (1039 mAh·g^–1^) after cycling, showing good reversibility and superior capacity to those of the other three anodes. Longer cycling and performance are shown in Figure 5c under 0.2 A·g^–1^. Initially, Si-SiO_*x*_/C, Si-SiO_*x*_/C-1.25, and Si-SiO_*x*_/C-2.5 show similar capacity (Figure 5a), while low initial capacity of Si-SiO_*x*_/C-5 is caused by denser structure with less access to inner Si inside structure. After several cycles, the capacities of Si-SiO_*x*_/C-1.25 and Si-SiO_*x*_/C-5 exhibit an increasing trend by access to more Si. Si-SiO_*x*_/C-1.25 exhibits the best cycling stability with a capacity retention of 74% at 630 mAh·g^–1^ after 300 cycles, while Si-SiO_*x*_/C degrades rapidly. GCD profiles of these four anodes after 10, 100, 200, and 300 cycles are shown in Figure S12, visually showing the capacity change during cycling. For Si-SiO_*x*_/C-5, more CNTs skeletons bring more pores inside the structure. With the continued volume expansion, more inner silicon should gradually be involved in the battery cycling, causing capacity to increase (10th and 100th cycle in Figure S12). However, the limited mechanical property (Figure S11) cannot withstand the volume expansion, resulting in a performance decay. Lower CNT content in Si-SiO_*x*_/C-2.5 with its denser structure improves the mechanical properties to some degree. However, more CNT loading in Si-SiO_*x*_/C-2.5 means that more silicon particles would be covered, reducing the number of electrochemically reactive sites. Combined with the fact that the mechanical properties are not sufficient to withstand the volume expansion upon lithiation, Si-SiO_*x*_/C-2.5 exhibits a reduced performance fade compared to that of Si-SiO_*x*_/C and the capacity is close to that of Si-SiO_*x*_/C after 300 cycles. For Si-SiO_*x*_/C-1.25, the optimal CNT loading attaches more C-PDA-Si and SiO_*x*_/C particles onto the skeleton, offering more electrochemically active sites with the capacity increasing in the first 50 cycles. The improved mechanical properties discussed previously (Figure 4d) can withstand volume expansion during cycling, exhibiting the best cycling performance.

EIS analyses after 100, 200, and 300 cycles were conducted to investigate the decay of capacity. The fitting of Si-SiO_*x*_/C after 300 cycles (Figure S13) consists of the resistances of the electrolyte solution (*R*_s_), the interfacial layer (*R*_suf_), and charge transfer (*R*_ct_). The evolution of resistances in these cells can be clearly seen in Figure 5d, Figure 5e, and Figure S14a and Figure S15a. Si-SiO_*x*_/C and Si-SiO_*x*_/C-2.5 increase intensely with the ongoing cycling process, potentially caused by intense volume expansion from the unstable structure, while the other two show a less increasing tendency, especially Si-SiO_*x*_/C-1.25. In Figure 5f, the resistances of the interfacial layer were summarized. With repeated lithiation and delithiation processes, the *R*_suf_ gradually increases, which is caused by the thickening of the SEI layer and volume expansion. However, curly CNTs can provide conductive pathways and buffer regions, reducing the resistance compared with Si-SiO_*x*_/C. Si-SiO_*x*_/C-1.25 with an optimal CNT content exhibits the lowest resistance, which is consistent with cycling performance. Compared with Si-SiO_*x*_/C-1.25, the significantly increased *R*_suf_ value of other Si-SiO_*x*_/Cs indicates that SEI layers are not stable due to repeated volume expansion. Repeated formation and destruction of the SEI layer consume lithium and electrolyte, resulting in the decay of battery performance.

**Figure 6:**
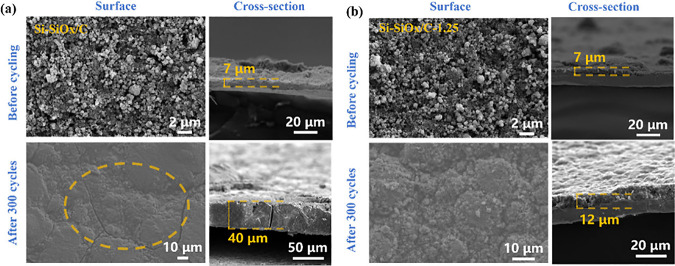
(a, b) SEM of surface and cross-section morphologies of Si-SiO_*x*_/C and Si-SiO_*x*_/C-1.25 before and after 300 cycles.

The morphologies of anodes before and after 300 cycles were observed via SEM to measure the volume expansion (Figure 6, Figure S14b, and Figure S15b). After 300 cycles, for Si-SiO_*x*_/C and Si-SiO_*x*_/C-2.5, cracks are obvious on the surface and delamination from the current collector occurred, which can explain the intense resistance increase (Figure 5f). Si-SiO_*x*_/C-5 has no obvious cracks but significant volume expansion, and Si-SiO_*x*_/C-1.25 shows uniform surface morphology and lower volume expansion. Based on the cross-section images before and after 300 cycles, the estimated volume expansion rates were listed in Table 2. The volume expansion of Si-SiO_*x*_/C-1.25 after 300 cycles is only 71.4%, which is much lower than the other three samples with 471.4% for Si-SiO_*x*_/C, 140.0% for Si-SiO_*x*_/C-2.5, and 206.7% for Si-SiO_*x*_/C-5, respectively. Therefore, the introduced CNT nanoskeleton structure could significantly reduce the negative effects of volume expansion and create more conductive pathways, thus improving cycling performance. Cycling performances under 0.5 A·g^–1^ (Figure S16a) and 1 A·g^–1^ (Figure S16b) were investigated. The performance decay under higher current densities shows a more intense tendency. Both the Si-SiO_*x*_/C and Si-SiO_*x*_/C-2.5 decay gradually during the repeated cycles and reach similar capacities after 300 cycles, caused by the volume expansion. Si-SiO_*x*_/C-5 showed the most stable performance under either two current densities, which could be caused by the buffering effect from the high contents of CNTs, while its capacity is not that high initially caused by the trapped Li^+^ in CNT.^38,39^ In summary, Si-SiO_*x*_/C-1.25 is still the optimal one. However, the performance Si-SiO_*x*_/C-1.25 under higher current density was not good enough like it was under lower current density, which could be caused by the conductivity limitation of the CNT nanoskeleton. Si-SiO_*x*_/C-1.25 delivers discharge capacities of 569 mAh·g^–1^ at 0.5 A·g^–1^ after 100 cycles and 293 mAh·g^–1^ at 1 A·g^–1^ after 300 cycles. Compared with the reported SiO_*x*_/C based anodes, Si-SiO_*x*_/C-1.25 reveals superior cycling performance and stability, which are attributed to the twined CNT skeleton structure and the ensuing conductive pathways between SiO_*x*_/C and carbon-wrapped silicon (Table S2).

**Table 2:** Thickness of Si-SiO_*x*_/Cs Anodes before and after 300 Cycles

	thickness before cycling (μm)	thickness after 300 cycles (μm)	volume expansion rate (%)
Si-SiO_*x*_/C	7	40	471.4
Si-SiO_*x*_/C-1.25	7	12	71.4
Si-SiO_*x*_/C-2.5	15	36	140.0
Si-SiO_*x*_/C-5	15	46	206.7

**Figure 7:**
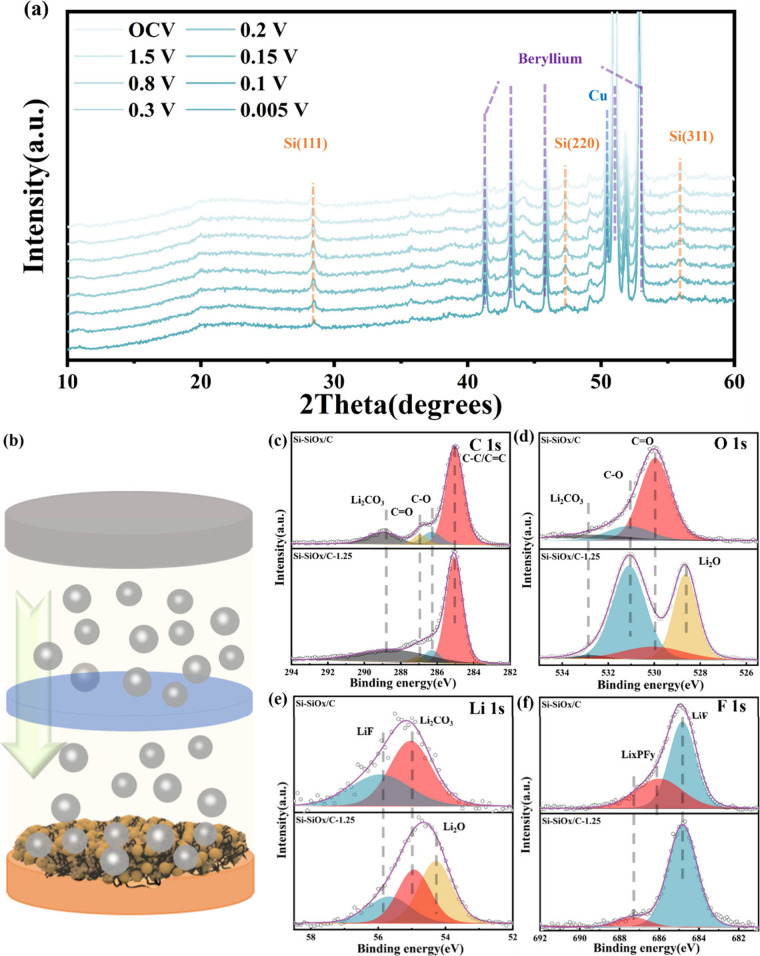
(a) In-operando XRD pattern of Si-SiO_*x*_/C-1.25 anode during initial lithiation process; (b) schematic diagram of Li deposition process on Si-SiO_*x*_/C-1.25 anodes; high-resolution XPS spectra of (c) C 1s, (d) O 1s, (e) Li 1s, and (f) F 1s in Si-SiO_*x*_/C and Si-SiO_*x*_/C-1.25 surface after full lithiation. XPS analysis of the SEI layer of Si-SiO_*x*_/C and Si-SiO_*x*_/C-1.25 is performed under 100 mA·g^–1^.

To further investigate the formation of the SEI layer and the properties, in-operando XRD analysis during the initial lithiation process was conducted. The cell was assembled in a specially designed split cell with a beryllium window on the top of the electrodes. In Figure 7a and Figure S17a, both the SiO_*x*_ and Si peaks of Si-SiO_*x*_/C and Si-SiO_*x*_/C-1.25 are clearly shown apart from peaks of copper and beryllium. No new peaks corresponding to Li–Si alloys can be observed during the lithium intercalation. The most obvious changes in Figure 7a are the intensity reduction and peak broadening of the Si (111), (220), and (311) reflections in the spectra. The normalized intensity of (111) is shown in Figure S17b and Figure S18, which show an obvious decrease upon lithiation. With more lithium intercalated into the silicon anodes, the intensity reduction and peak broadening become more severe, indicating that the lithiation process converted silicon in the crystal state into an amorphous state or lithium-Si alloys on the nanoscale, which cannot be detected by XRD.^40^

To further analyze the components of SEI layer, the XPS spectra were collected when lithium is fully intercalated into the anodes under 0.1 A·g^–1^, as illustrated in Figure 7b. For comparison, XPS analysis of anodes before cycling was also conducted, as shown in Figure S19. The XPS spectra before cycling show the Si 2s and Si 2p peaks from SiO_*x*_/C and Si nanoparticles, while Si related peaks disappear after full lithiation, indicating that the outer SEI layer covers the anode surface and has enough thickness to stop the Si signal.^41^ Also, both the Si-SiO_*x*_/C and Si-SiO_*x*_/C-1.25 show the existence of F, C, and Li elements, verifying the formation of the SEI layer. From the high-resolution C 1s and O 1s spectra (Figure 7c, Figure 7d), the C–O is detected at 286.3 and 531.1 eV while the C=O is detected at 286.9 and 530.0 eV,^42^ which stems from the reduction of the electrolyte. The peaks at 289.0 eV in C 1s, 532.9 eV in O 1s, and 55.1 eV in Li 1s (Figure 7e) are related to Li_2_CO_3_.^43^ It is noted that the signal of Li_2_CO_3_ in Si-SiO_*x*_/C-1.25 is weaker than that in Si-SiO_*x*_/C, indicating that the SEI layer on the surface of Si-SiO_*x*_/C has the lower content of Li_2_CO_3_. In F 1s (Figure 7f), the peaks ranging from 686 to 688 eV are related to Li_*x*_PF_y,_ which can further be further reduced to LiF, while the corresponding peaks of LiF are at 55.8 eV in Li 1s and 685 eV in F 1s. Moreover, another main difference of O 1s and Li 1s between Si-SiO_*x*_/C and Si-SiO_*x*_/C-1.25 is the existence of peaks related to Li_2_O in Si-SiO_*x*_/C-1.25, which are located at 528.6 eV in O 1s and 54.2 eV in Li 1s.^42−44^ Hence, based on the XPS results, the SEI layer of Si-SiO_*x*_/C-1.25 contains lower content of Li_2_CO_3_ and Li_2_O, which Si-SiO_*x*_/C does not have. With the presence of excess lithium atoms from the counter electrode, electrolyte decomposes to form Li_2_CO_3_ and Li_2_O. Furthermore, the obtained Li_2_CO_3_ is not stable and continues to form Li_2_O.^45^ These processes produce CO_2_ increasing the porosity of anodes and thicken the SEI layer, causing the decay of battery performance. The low content of Li_2_CO_3_ on the surface of Si-SiO_*x*_/C-1.25 reduces the following decomposition to Li_2_O and increases the stability of SEI layer, forming denser and more homogeneous SEI layer. Hence, the stable SEI layer could prevent the continuous side reactions between anodes and electrolyte. Moreover, the evenly introduced CNT nanoskeleton structure can improve the distribution of conductivity pathways in Si-SiO_*x*_/C-1.25 as shown in CAFM (Figure 4). The homogeneous electric contact of particles in Si-SiO_*x*_/C-1.25 with electrolyte can promote the formation of thin and compact SEI layer.^46^

**Figure 8:**
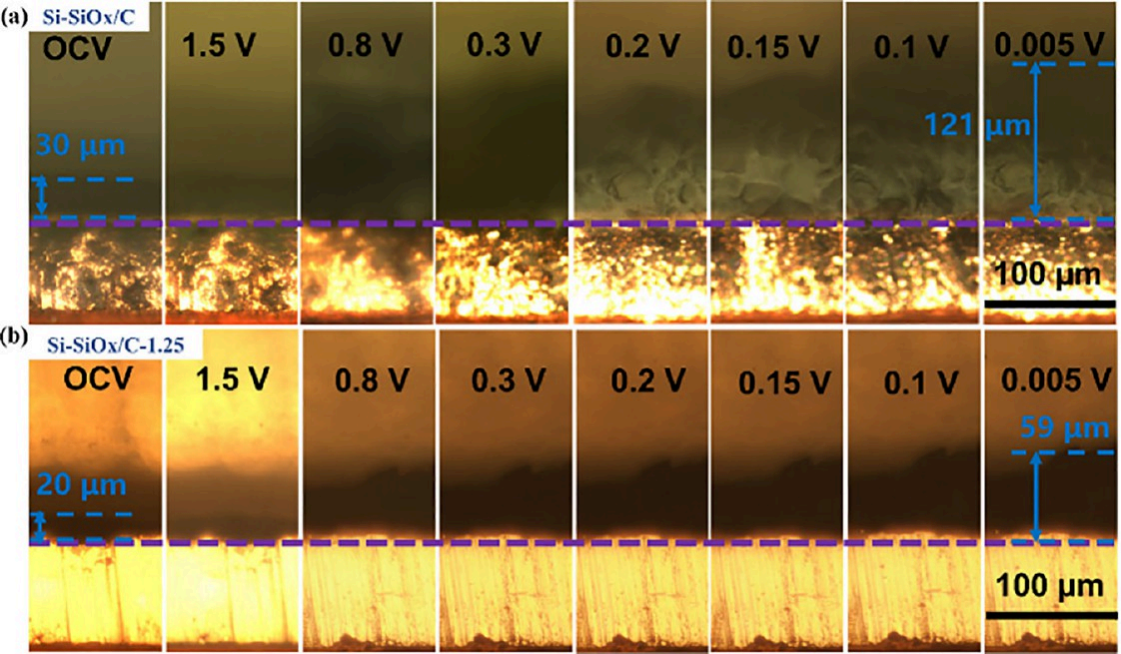
In-operando optical figures for cross-section morphologies of (a) Si-SiO_*x*_/C anode and (b) Si-SiO_*x*_/C-1.25 anode.

Optical cells were assembled to observe the SEI formation and volume expansion in-operando during the initial lithiation process. In Figure 8a and Figure 8b, the thickness of Si-SiO_*x*_/C increases significantly from 30 to 121 μm, while the structure of Si-SiO_*x*_/C-1.25 is more stable, and the thickness only increases from 20 to 59 μm with a clearly denser structure. This denser structure indicates that the CNT nanoskeleton can reduce the damage of SEI layer during cycling. On the other hand, superior mechanical properties and buffer regions enabled by the CNTs can significantly reduce the volume expansion of silicon, avoiding the destruction of the SEI layer on the surface. The stable SEI layer prevents unnecessary side reactions, avoiding the continuous consumption of electrolytes. Therefore, combining with the improved Young’s modulus, improved conductive pathways, and stable SEI layer, CNT supported nanoskeleton Si-SiO_*x*_/C-1.25 provides superior battery cycling performance.

**Figure 9:**
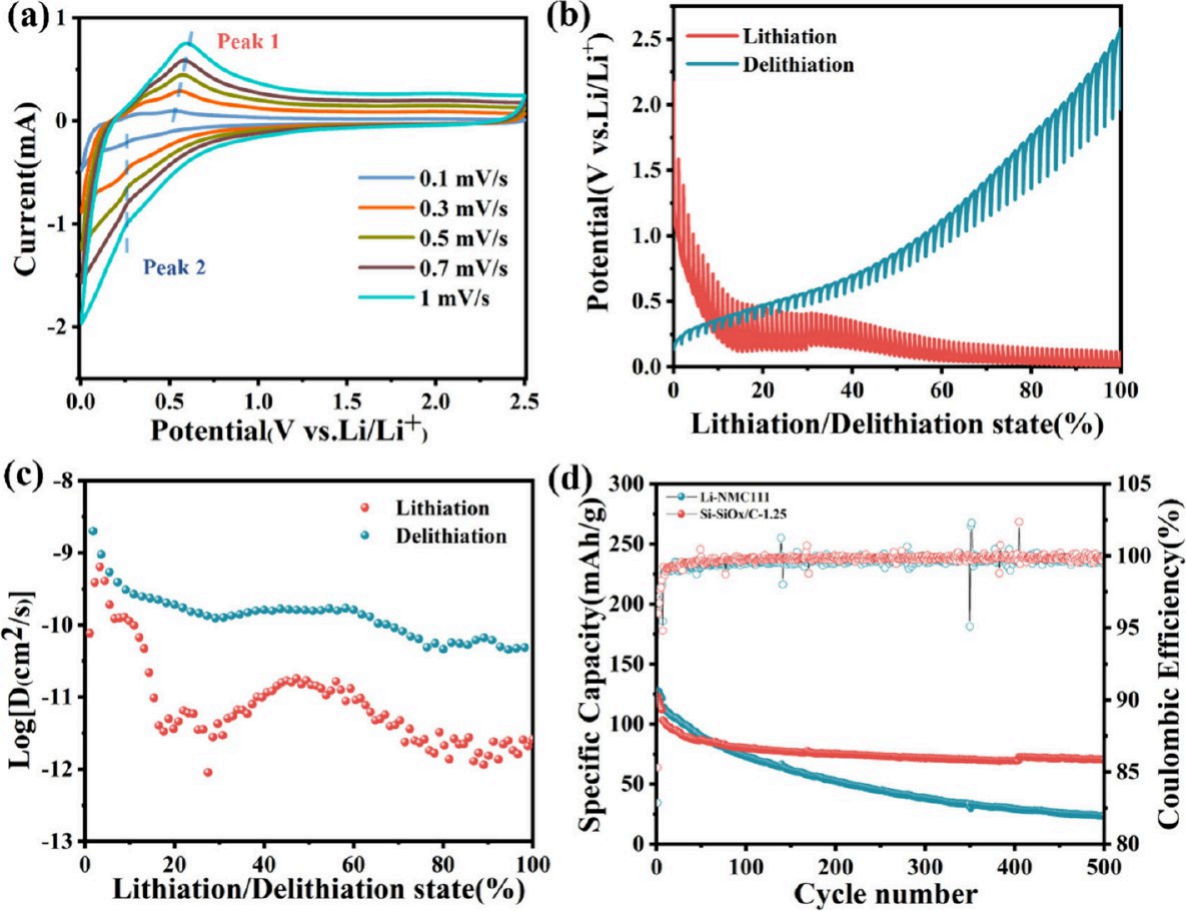
(a) CV curves of Si-SiO_*x*_/C-1.25 anode at different scan rates; (b) GITT profiles of Si-SiO_*x*_/C-1.25 anode; (c) *D*_Li+_ of Si-SiO_*x*_/C-1.25 anode calculated from GITT profiles; (d) cycling performance and Coulombic efficiency of Li metal-NMC111 and Si-SiO_*x*_/C-1.25-NMC111 full cell under 100 mA·g^–1^ with the first five cycles at 50 mA·g^–1^ as activation process.

Before application into full cell structure, (de)intercalation kinetics of Si-SiO_*x*_/C-1.25 were analyzed. The CV at different scan rates (0.1–1 mV·s^–1^) were collected. In Figure 9a, the peak currents (*i*) increase with the increase of scan rate (*v*), based on the power-law equation:^47^
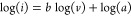
1In the eq 1, the *b* value is used to determine the Li^+^-diffusion controlled behavior when *b* = 0.5 and pseudocapacitive-controlled behavior when *b* = 1.^48^ For Si-SiO_*x*_/C-1.25, the obtained *b* values for the anodic peak (peak 1) and cathodic peak (peak 2) are 0.90 and 0.70, respectively, indicating the mixed Li^+^ storage process (Figure S20).

The diffusion coefficient of Li^+^ (*D*_Li+_) in Si-SiO_*x*_/C-1.25 was further calculated via the galvanostatic intermittent titration technique (GITT) with the titration time and rest time of 10 min and 2 h at 100 mA·g^–1^ as shown in Figure 9b. *D*_Li+_ can be calculated by the following equation:
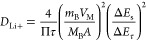
2where the τ, *V*_M_, *m*_B_, *M*_B_, *A*, Δ*E*_s_, and Δ*E*_τ_ represent the titration time, the molar volume, the mass and molar mass of active materials, the surface area of the electrode, the voltage changes within the rest time, and the voltage changes within the titration time, respectively. Figure 9c shows the calculated *D*_Li+_ values during lithiation and delithiation processes based on eq 2. The value locates in the range of 10^–8^ to 10^–12^ cm^2^·s^–1^, higher than *D*_Li+_ from some literature.^49,50^ The result confirms the fast Li^+^ diffusion in Si-SiO_*x*_/C-1.25, indicating the advantage of nanoskeleton structure for enhancing the electrochemical performance.

To investigate the performance of Si-SiO_*x*_/C-1.25 in a full cell, the prelithiated Si-SiO_*x*_/C-1.25 was used to pair with a NMC111 cathode to assemble a full cell. As shown in Figure 9d, the full cell is cycled at 100 mA·g^–1^ with a full cell consisting of lithium metal and NMC111 as a control sample. The first five cycles are 50 mA·g^–1^ as an activation process. The initial capacity of the Li metal-NMC111 cell is higher than that of Si-SiO_*x*_/C-1.25. However, the Si-SiO_*x*_/C-1.25-NMC111 cell remains stable and even reaches 71 mAh·g^–1^ after 500 cycles with a capacity retention of 72%, exhibiting excellent cycling stability, while the Li metal-NMC111 cell drops to 24 mAh·g^–1^.

## 4 Conclusions

In summary, CNT supported “nanoskeleton” Si-SiO_*x*_/C anodes were fabricated by an in situ cross-linking reaction between GA and APTMS with the presence of polydopamine-wrapped silicon nanoparticles and CNTs. The CNT nanoskeleton can improve electrical contact between particles and increase conductive pathways, as confirmed by CAFM, as well as promoting the formation of a denser and homogeneous SEI layer. Curly CNTs can also work as buffer regions and improve the mechanical property (Young’s modulus), better withstanding the volume expansion of Si. Therefore, the repeated destruction of the SEI layer during continuous cycling can be avoided. The stable and dense SEI layers are formed on the surface of Si-SiO_*x*_/C-1.25, observed by in-operando XRD, optical microscope, and ex situ XPS. The Si-SiO_*x*_/C-1.25 anode delivered a high capacity (918 mAh·g^–1^ at 200 mA·g^–1^) and high-capacity retention (74% after 300 cycles) with reduced volume expansion (71.4%). With the stabilized SEI layer and high capacity of the Si-SiO_*x*_/C-1.25 anode, the specific capacity of the NMC111 full cell is stable at about 71 mAh·g^–1^ after 500 cycles at 100 mA·g^–1^ with a capacity retention of 72%. These results suggest that this “nanoskeleton” anode supported by CNTs has great potential to improve cycling stability of Si anode in full cell.

## Supporting Information Available

FT-IR of dopamine chloride and PDA-Si, Raman spectra of PDA-Si, XPS spectra of P-Si-SiO_*x*_/Cs, Si 2p and O 1s XPS spectra of P-Si-SiO_*x*_/Cs, SEM images of carbonized APTMS, STEM images and relevant EDS mapping, XPS spectra of Si-SiO_*x*_/Cs, XRD pattern of Si-SiO_*x*_/Cs, O 1s XPS spectra of Si-SiO_*x*_/Cs, AFM, CAFM images and relevant Young’s modulus distribution, GCD profiles of Si-SiO_*x*_/Cs after fixed cycles, Nyquist plot of EIS spectra and equivalent circuit model, EIS spectra of Si-SiO_*x*_/Cs after fixed cycles and SEM of anodes after 300 cycles, cycling performance and Coulombic efficiency at 0.5 A·g^–1^ and 1 A·g^–1^, in-operando XRD pattern of Si-SiO_*x*_/C anode during initial lithiation process and normalized intensity of Si (111) at different voltages, normalized intensity of Si (111) in Si-SiO_*x*_/C-1.25 anode from XRD at different voltages, XPS spectra of Si-SiO_*x*_/C anode and Si-SiO_*x*_/C-1.25 anode before and after lithiation, log(*i*) versus log(*v*) plots of the cathodic and anodic peaks for Si-SiO_*x*_/C-1.25, average Si valence state of Si-SiO_*x*_/Cs, and comparison of Si-SiO_*x*_/C composites in reported literature to this work (PDF)
