# Supplementary material for: “Nanoskeleton” Si-SiOx/C Anodes toward Highly Stable Lithium-Ion Batteries
Source: ACS Appl Mater Interfaces. 2025 Feb 4;17(7):10580–92. doi: 10.1021/acsami.4c18254 (PMC11843606; doi:10.1021/acsami.4c18254)
Supplement: Supplementary file 1 — am4c18254_si_001.pdf [file am4c18254_si_001.pdf]

Supporting Information

“Nano-Skeleton” Si-SiO$_x$/C Anodes towards Highly Stable Lithium-Ion Batteries

Xiang Guan, Yang Zhang, Ian A. Kinloch, Mark A. Bissett*
National Graphene Institute, Henry Royce Institute, and Department of Materials
University of Manchester, Manchester M13 9PL, UK
E-mail: mark.bissett@manchester.ac.uk

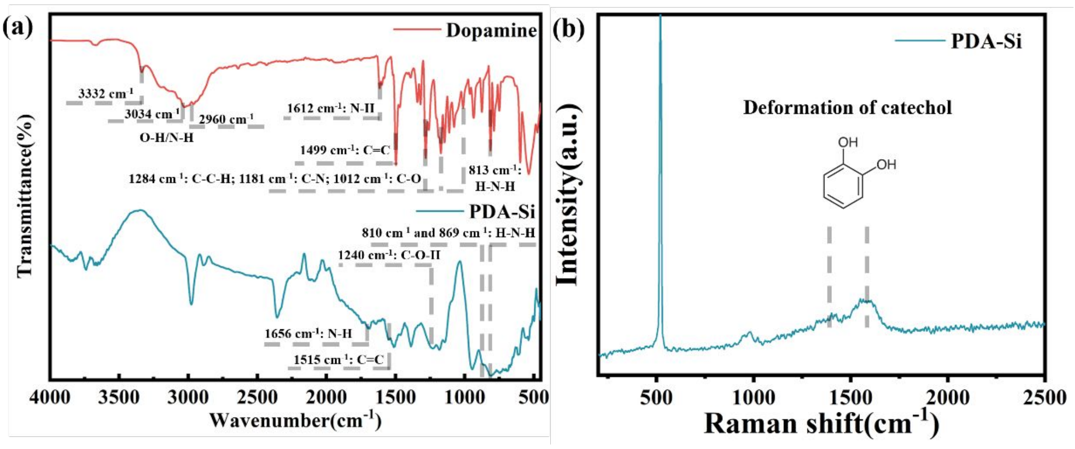

Figure S1 (a) FT-IR of dopamine chloride and PDA-Si; (b) Raman spectra of PDA-Si.

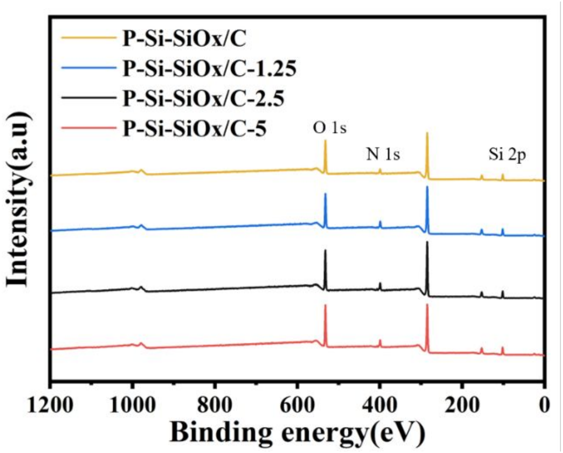

Figure S2 XPS spectra of P-Si-SiO$_x$/Cs.

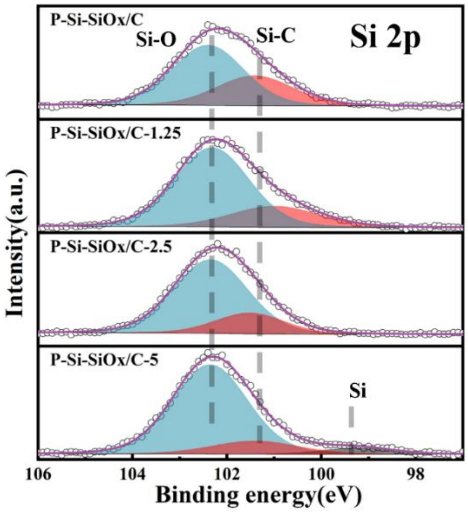

Figure S3 Si 2p XPS spectra of P-Si-SiO_x/Cs.

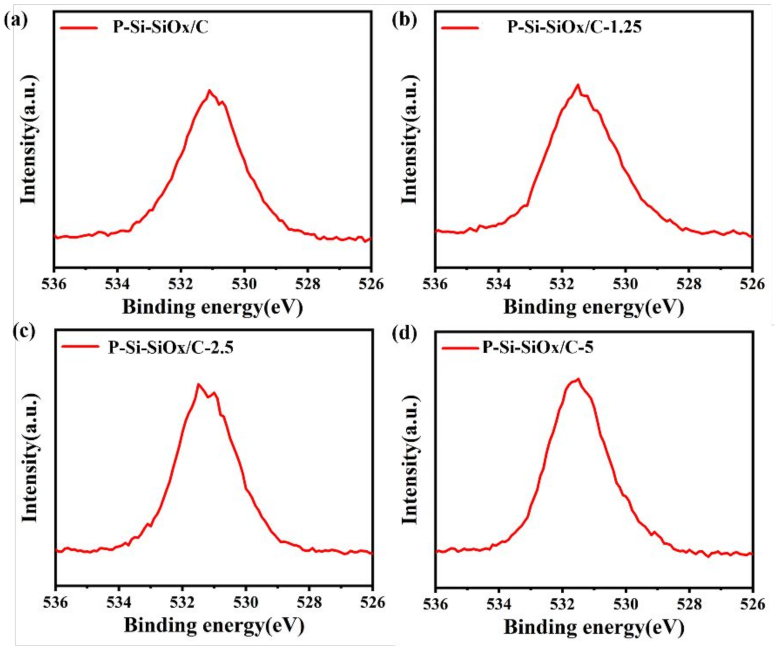

Figure S4 O 1s XPS spectra of P-Si-SiO_x/Cs: (a) P-Si-SiO_x/C, (b) P-Si-SiO_x/C-1.25, (c) P-Si-SiO_x/C-2.5, (d) P-Si-SiO_x/C-5.

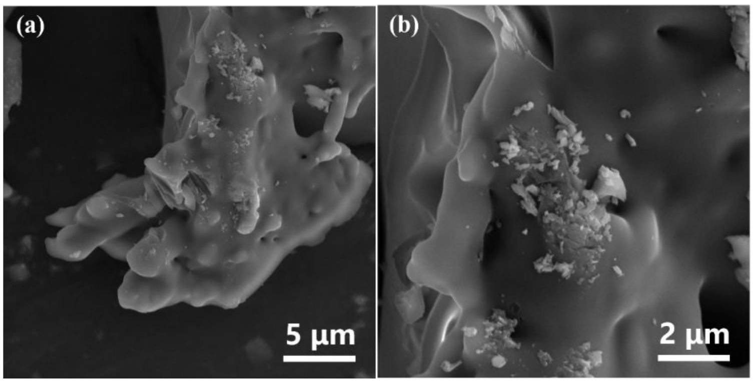

Figure S5 (a), (b) SEM images of carbonized APTMS.

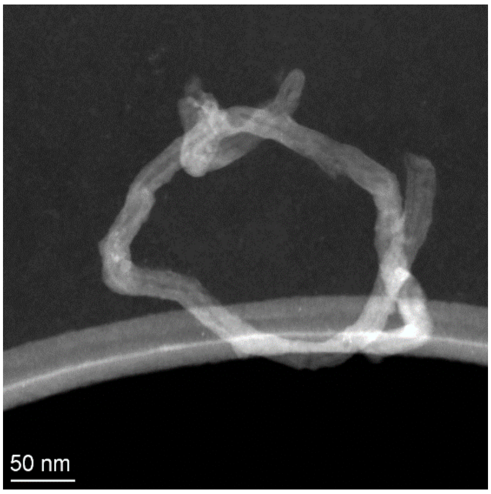

Figure S6 STEM image of CNTs.

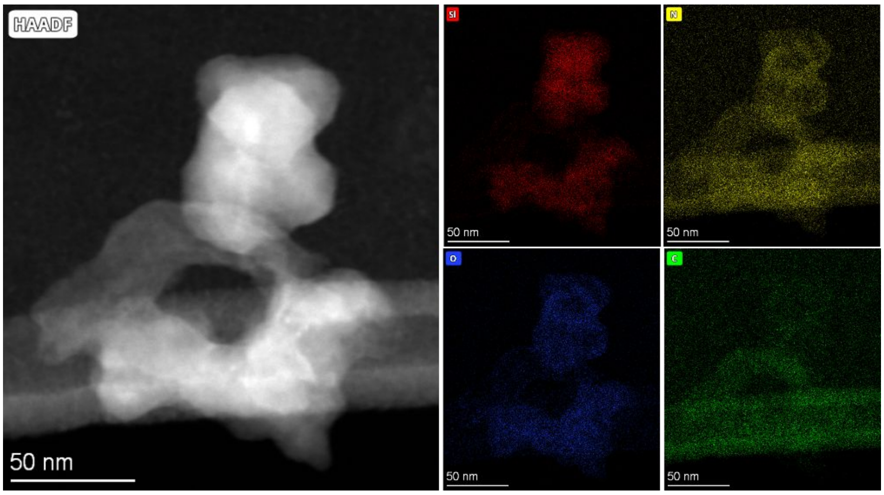

Figure S7 STEM image and related EDS mapping of Si-SiO$_x$/C-1.25.

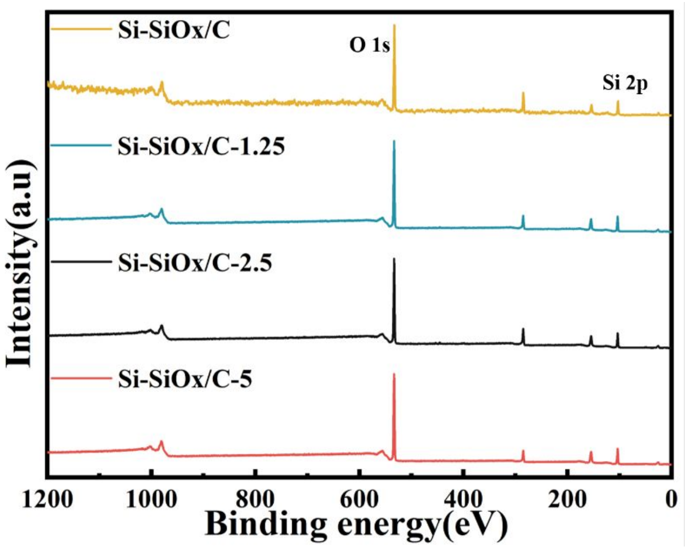

Figure S8 XPS spectra of Si-SiO$_x$/Cs.

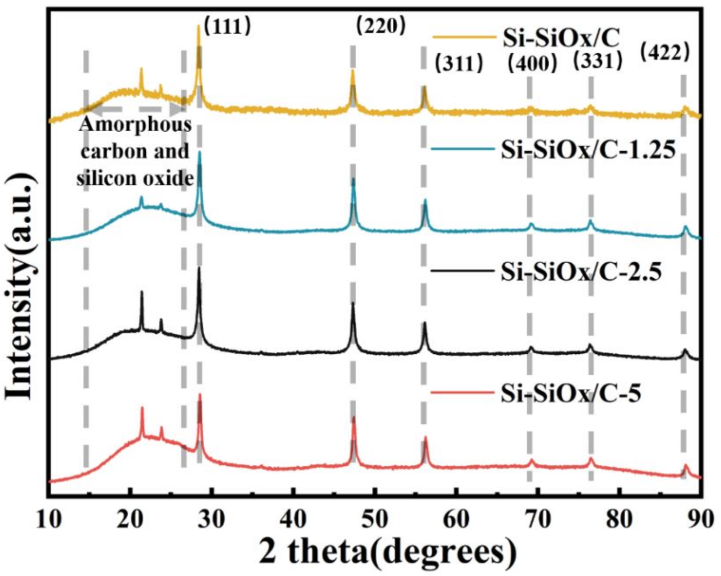

Figure S9 XRD pattern of Si-SiO_x/Cs.

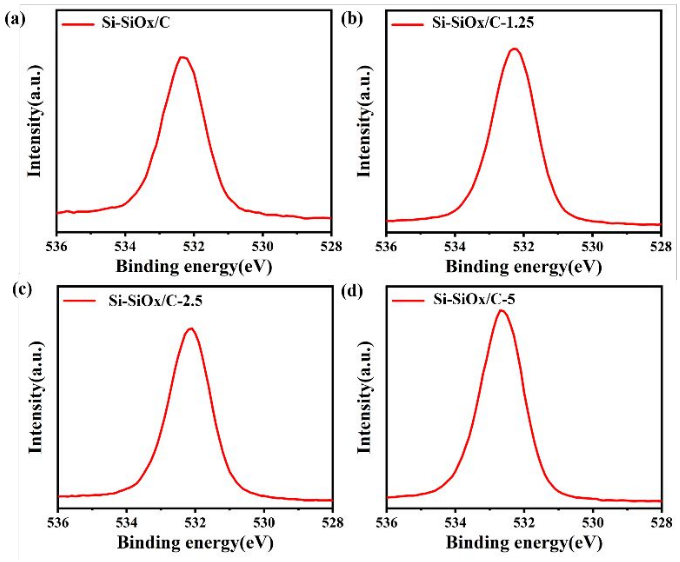

Figure S10 O 1s XPS spectra of Si-SiO_x/Cs: (a) Si-SiO_x/C, (b) Si-SiO_x/C-1.25, (c) Si-SiO_x/C-2.5, (d) Si-SiO_x/C-5.

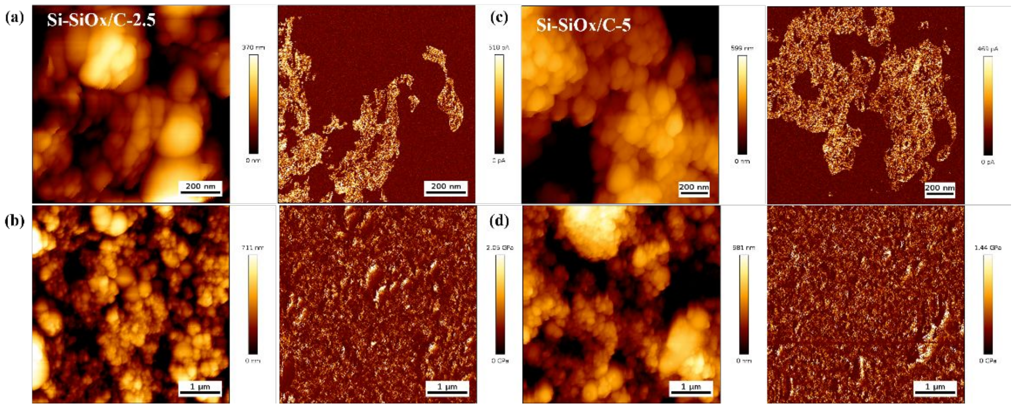

Figure S11 (a) AFM and CAFM images of Si-SiO_x/C-2.5; (b) AFM image and Young's modulus distribution of Si-SiO_x/C-2.5; (c) AFM and CAFM images of Si-SiO_x/C-5; (d) AFM image and Young's modulus distribution of Si-SiO_x/C-5.

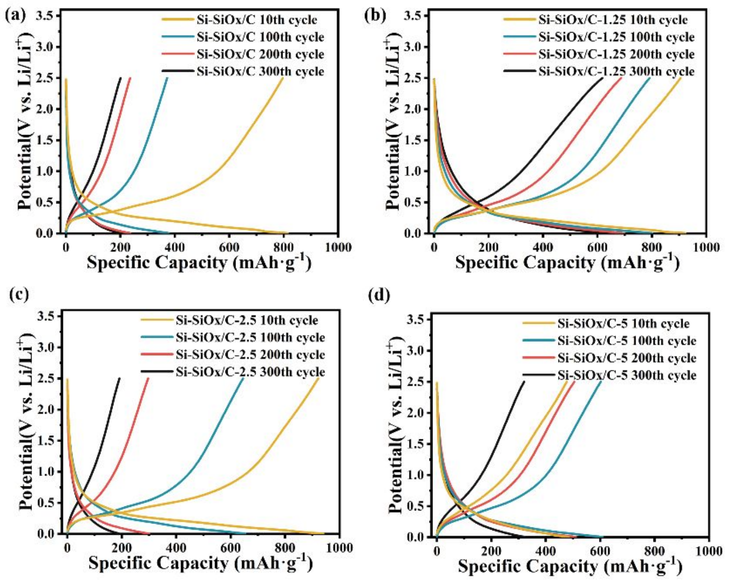

Figure S12 GCD profiles of Si-SiO_x/Cs after 10, 100, 200 and 300 cycles: (a) Si-SiO_x/C, (b) Si-SiO_x/C-1.25, (c) Si-SiO_x/C-2.5, (d) Si-SiO_x/C-5.

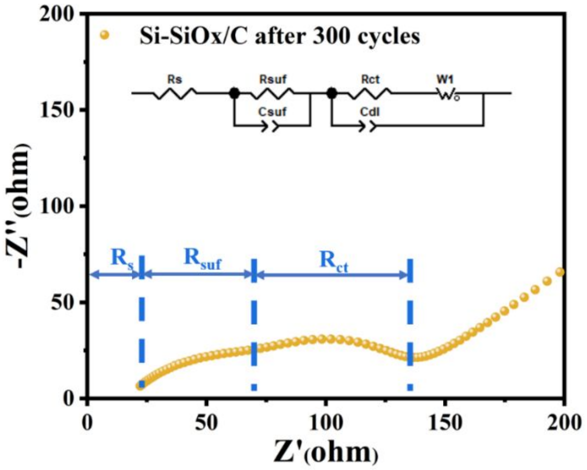

Figure S13 Nyquist plot of EIS spectra for Si-SiO_x/C measured after 300 cycles (inset is the equivalent circuit model).

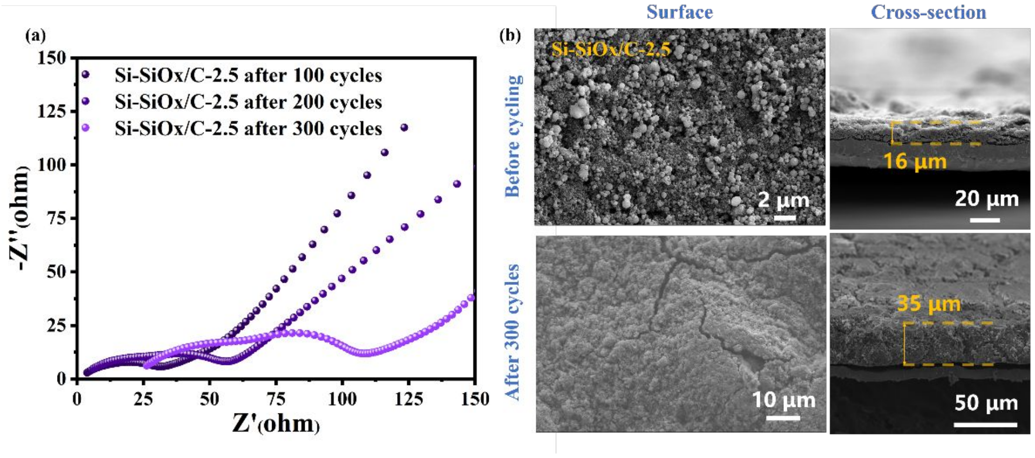

Figure S14 (a) EIS spectra of Si-SiO_x/C-2.5 after 100, 200 and 300 cycles at 0.2 $A \cdot g^{-1}$; (b) SEM of surface and cross-section morphologies of Si-SiO_x/C-2.5 before and after 300 cycles.

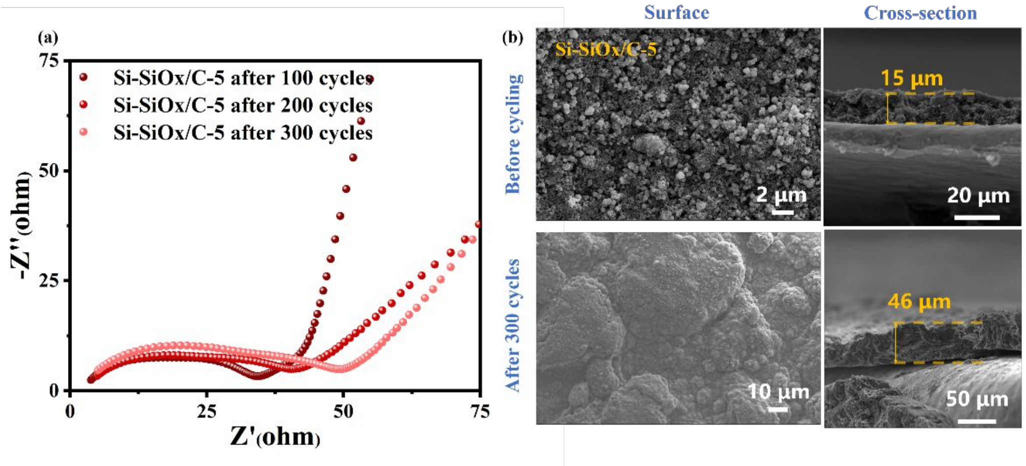

Figure S15 (a) EIS spectra of Si-SiO_x/C-5 after 100, 200 and 300 cycles at 0.2 A·g^{-1}; (b) SEM of surface and cross-section morphologies of Si-SiO_x/C-5 before and after 300 cycles.

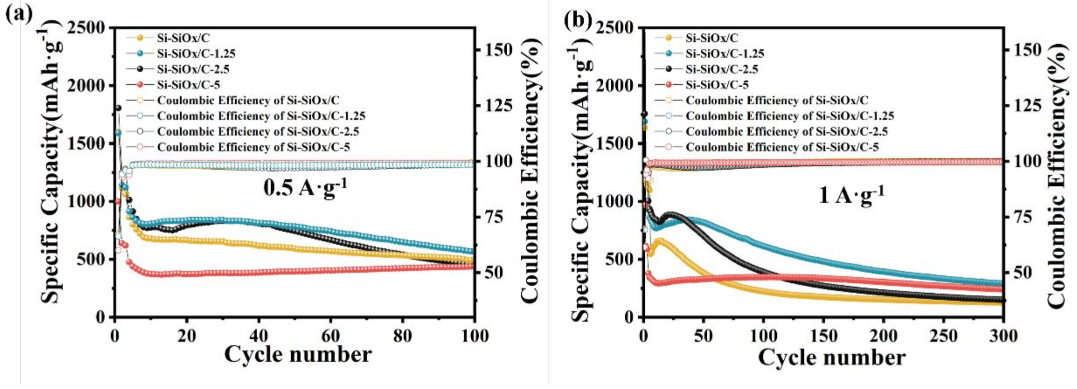

Figure S16 (a) Cycling performance and coulombic efficiency at 0.5 A·g^{-1} with the first five cycles at 0.1 A·g^{-1} as activation process; (b) Cycling performance and coulombic efficiency at 1 A·g^{-1} with the first five cycles at 0.1 A·g^{-1} as activation process.

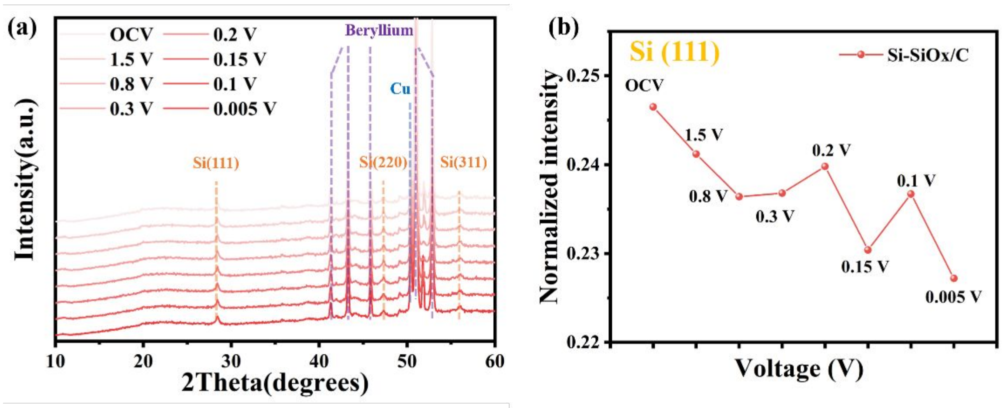

Figure S17 (a) In-operando XRD pattern of Si-SiO_x/C anode during initial lithiation process; (b) normalized intensity of Si (111) in Si-SiO_x/C anode from XRD at different voltages.

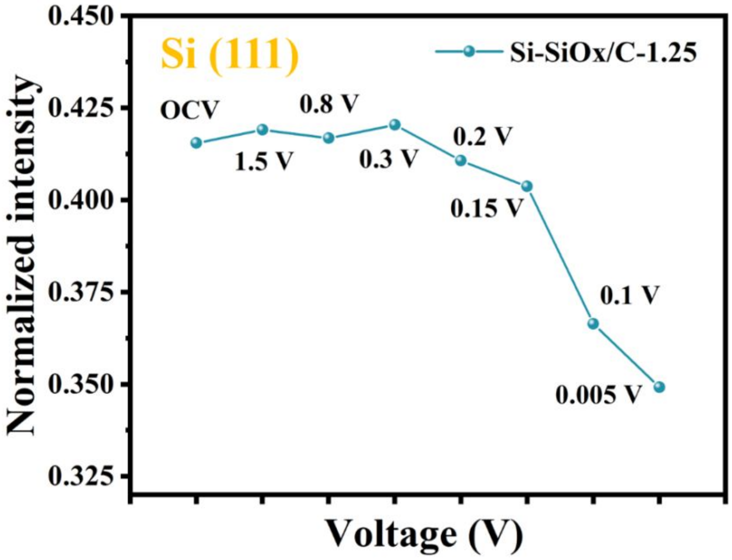

Figure S18 Normalized intensity of Si (111) in Si-SiO$_x$/C-1.25 anode from XRD at different voltages.

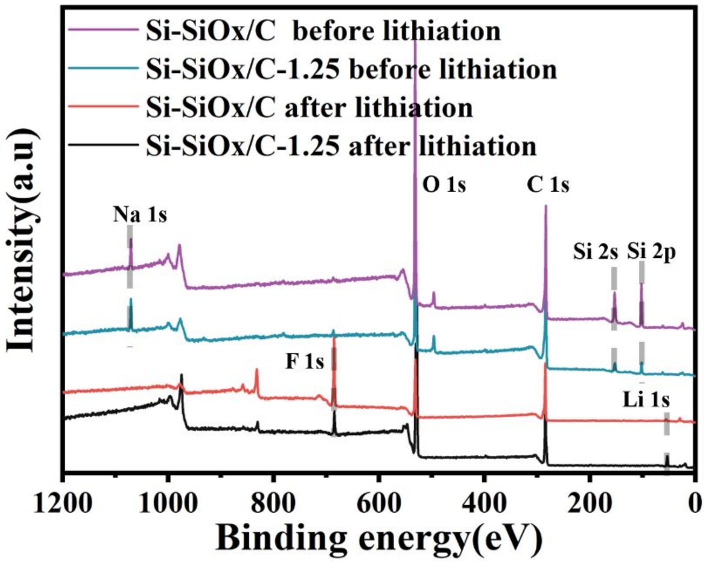

Figure S19 XPS spectra of Si-SiO$_x$/C anode and Si-SiO$_x$/C-1.25 anode before and after lithiation.

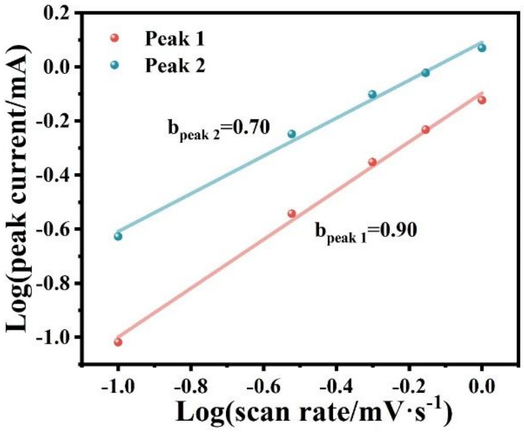

Figure S20 Log(i) versus log(v) plots of the cathodic and anodic peaks for Si-SiO$_x$/C-1.25 anode.

Table S1 Average Si valence state of Si-SiO_x/Cs materials

Sample ID	Si^{2+}	Si^{3+}	Average Si valence state
Si-SiO_x/C	48.01%	51.99%	3.56
Si-SiO_x/C-1.25	85.87%	14.13%	2.14
Si-SiO_x/C-2.5	86.39%	13.61%	2.14
Si-SiO_x/C-5	72.42%	27.58%	2.28

Table S2 Comparison of Si-SiO_x/C composites in reported literature to this work

Sample	Structure	Capacity (mAh·g^{-1})	Ref.
SiOC/nSi	SiO_x@C/nano-silicon composites	600 (74 mA·g^{-1}, 100 cycles)	1
SiOC	SiO_x@C	800 (200 mA·g^{-1}, 250 cycles)	2
SiOC-P2	SiO_x@C with nanovoids	~770(360 mA·g^{-1}, 200 cycles)	3
SiO_x@C	nanorods	720 (100 mA·g^{-1}, 350 cycles)	4
Si-SiO_x/C-1.25	CNT supported skeleton	630 (200 mA·g^{-1}, 300 cycles)	This work

Supporting References

(1) Kaspar, J.; Graczyk-Zajac, M.; Lauterbach, S.; Kleebe, H. J.; Riedel, R. Silicon Oxycarbide/Nano-Silicon Composite Anodes for Li-Ion Batteries: Considerable Influence of Nano-Crystalline vs. Nano-Amorphous Silicon Embedment on the Electrochemical Properties. *J Power Sources* **2014**, *269*, 164–172.

(2) Halim, M.; Hudaya, C.; Kim, A. Y.; Lee, J. K. Phenyl-Rich Silicone Oil as a Precursor for SiOC Anode Materials for Long-Cycle and High-Rate Lithium Ion Batteries. *J Mater Chem A Mater* **2016**, *4* (7), 2551–2556.

(3) Lee, S. H.; Park, C.; Do, K.; Ahn, H. Maximizing the Utilization of Active Sites through the Formation of Native Nanovoids of Silicon Oxycarbide as Anode Materials in Lithium-Ion Batteries. *Energy Storage Mater* **2021**, *35*, 130–141.

(4) Ren, Y.; Li, M. Facile Synthesis of SiOx@C Composite Nanorods as Anodes for Lithium Ion Batteries with Excellent Electrochemical Performance. *J Power Sources* **2016**, *306*, 459–466.
